# Supplementary material for: Structural basis for species-selective targeting of Hsp90 in a pathogenic fungus
Source: Nat Commun. 2019 Jan 24;10:402. doi: 10.1038/s41467-018-08248-w (PMC6345968; doi:10.1038/s41467-018-08248-w)
Supplement: Supplementary file 1 — Supplementary Information [file 41467_2018_8248_MOESM1_ESM.pdf]

Supplementary Information

Structural basis for species-selective targeting of Hsp90 in a pathogenic fungus
Whitesell et al.

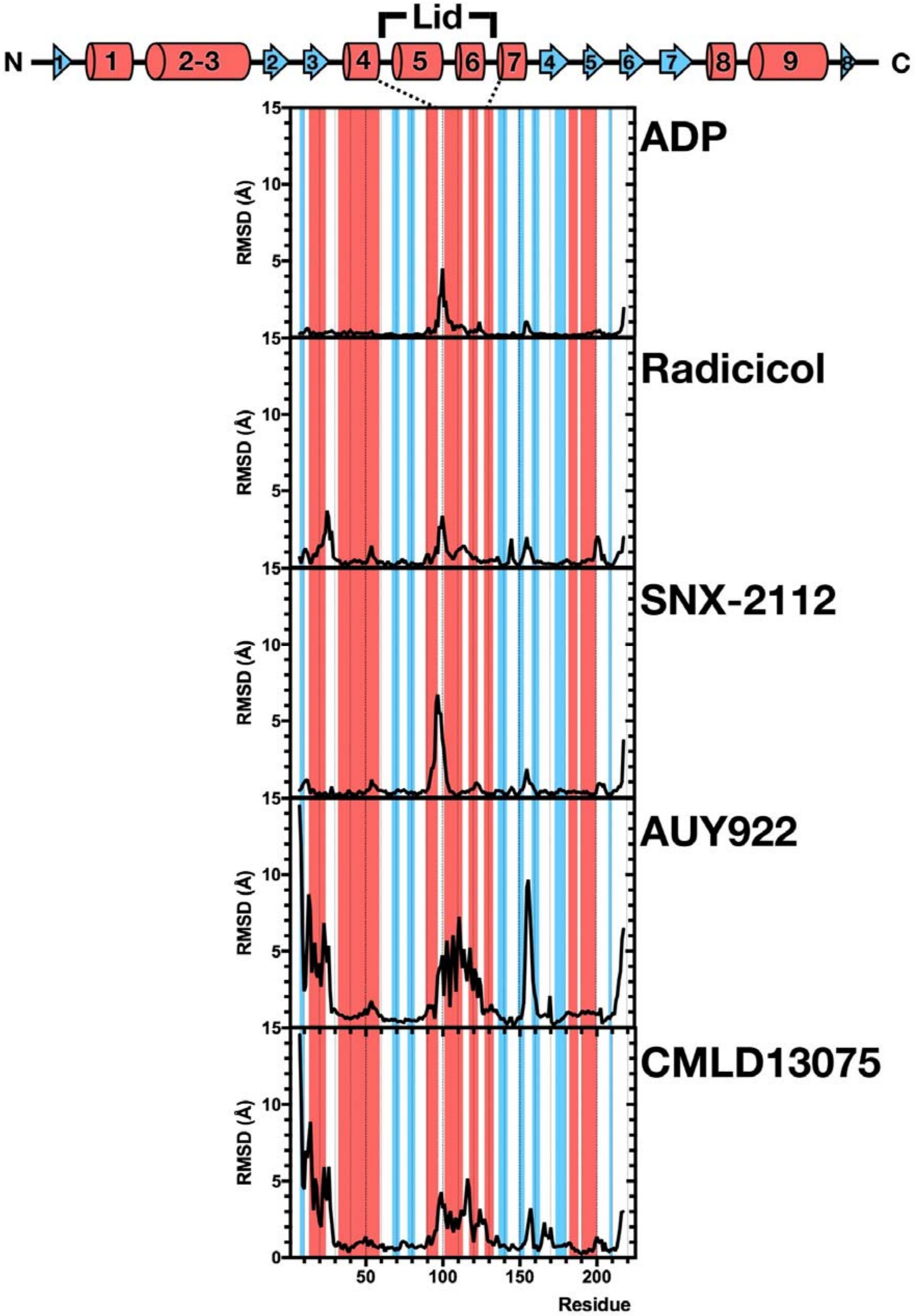

Supplementary Figure 1

Supplementary Figure 1

RMSD differences observed for *C. albicans* NBD co-crystals. Plot of the average r.m.s.d. observed per residue for main-chains atoms upon comparing the structure of ligand-free *C. albicans* Hsp90 NBD with all the protein-ligand co-crystal structures reported here. The color shading represents the position of secondary structural elements in the *C. albicans* Hsp90 NBD *apo* structure; β-strands (blue) and α-helices (red) as depicted at the top of the graph. The location and structural composition of the lid subdomain is indicated.

Biochemical characterization of *C. albicans* Hsp90

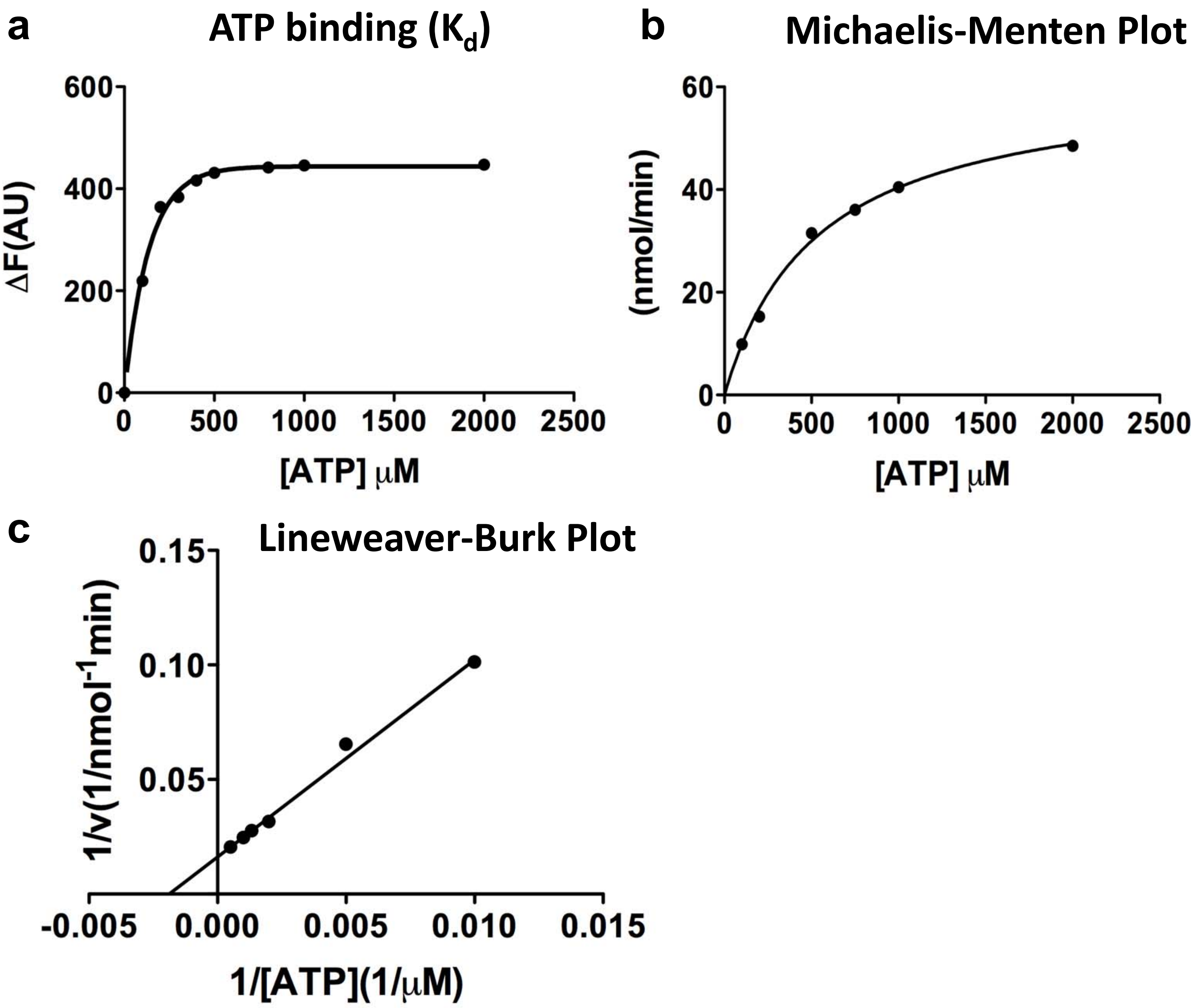

d Comparison Summary

Organism	K_m (μM)	Kcat (min^{-1})	kcat/K_m ($min^{-1}μM^{-1}$)	K_d (μM)
C. albicans	533	6.2 X 10^{-2}	11.6 X 10^{-5}	125
S. cerevisiae[a]	511	8.0 X 10^{-2}	15.6 X 10^{-5}	132
Human[a]	324	1.5 X 10^{-2}	4.6 X 10^{-5}	240

[a] Data from: Owen, B. A. L. et al. Regulation of heat shock protein 90 ATPase activity by sequences in the carboxyl terminus. *J. Biol. Chem.* **277,** 7086–7091 (2002)

Supplementary Figure 2

Supplementary Figure 2

Biochemical characterization of *C. albicans* Hsp90. a, Binding of *C. albicans* Hsp90 to its natural ligand ATP. **b,** Michaelis-Menten plot correlating the rate of ATP hydrolysis by *C. albicans* Hsp90 with concentration of substrate. Fractional cleavage of γ-^{32}P-labeled ATP is plotted against ATP concentration. **c,** Lineweaver-Burk plot performed to define K_m and kcat for the ATPase activity of *C. albicans* Hsp90. **d,** Comparison of parameters determined for *C. albicans* Hsp90 with previously published data for homologous human and yeast (*S. cerevisiae*) proteins.

a Affinity of fluorescence polarization (FP) probe for nucleotide binding domains (NBD)

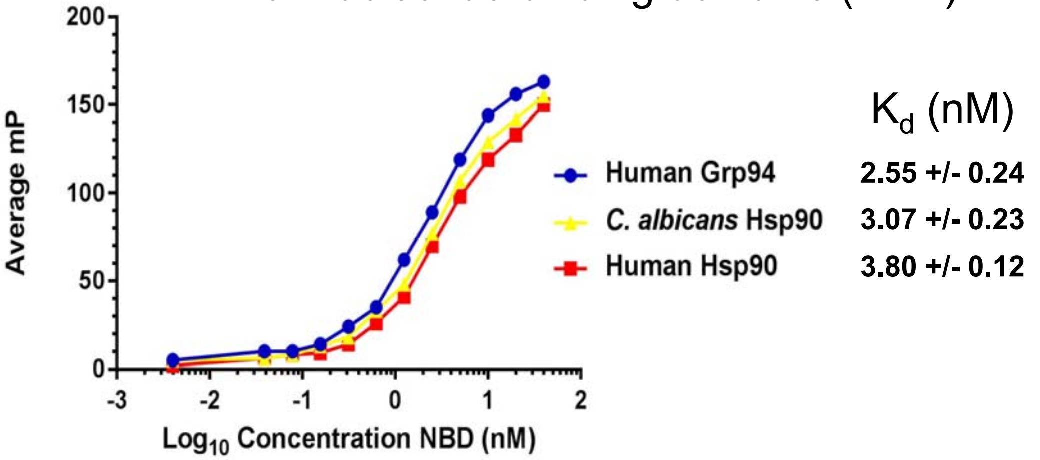

b Affinity of inhibitors for nucleotide binding domains (K_i, nM)

Compound	Human Hsp90	Candida Hsp90	Human Grp94
**geldanamycin**	**4.9 +/-0.51**	**5.6 +/- 0.66**	**4.1 +/- 0.29**
**AUY922**	**<0.1**	**0.2 +/-0.03**	**0.9 +/-0.04**
**SNX-2112**	**0.9 +/-0.03**	**0.3 +/-0.05**	**63.8 +/-8.40**

c Relative inhibitor potency in whole cell lysates by FP assay

**FP EC_{50} (nM)**	geldanamycin (GdA)	AUY922	SNX-2112
Human	**18.9 +/- 0.95**	**18.7 +/- 0.51**	**92.0 +/- 7.73**
C. albicans	**18.9 +/- 0.95**	**11.2 +/- 0.17**	**11.5 +/- 0.48**

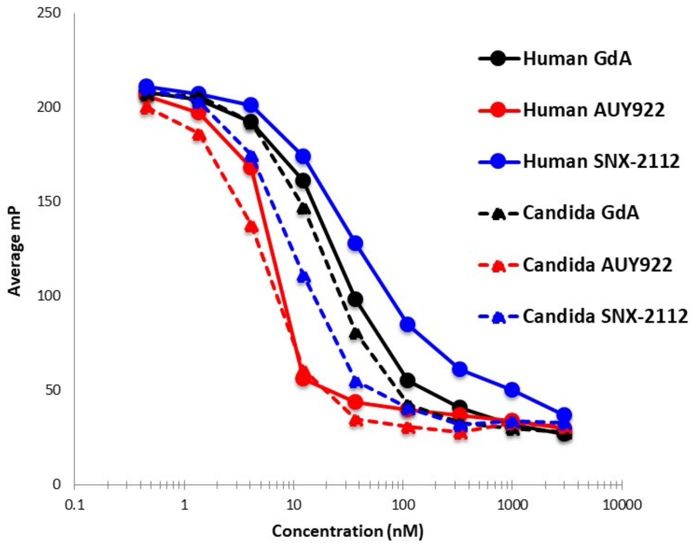

Representative equilibrium binding displacement curves from a single FP experiment.

Supplementary Figure 3

Supplementary Figure 3

Determination of NBD binding affinities by FP. a, Saturation binding of fluorescent probe (Cy3-geldanamycin) to purified NBDs derived from human Grp94 and Hsp90 as well fungal Hsp90 was measured as a decrease in millipolarization (mP) units. The anisotropy of a fixed concentration of probe (0.1 nM) was measured over a range of NBD concentrations to determine IC_{50} values by variable slope 4-parameter curve fitting of duplicate determinations at each concentration tested (R^2>0.98, all curves). K_d values were calculated from these data using previously published methods. FP assays were repeated in 3 independent experiments. The mean +/- SEM is presented. **b,** Equilibrium competition binding FP assays were performed with purified NBDs to determine the K_i of inhibitors using the probe dissociation constants defined in panel **a** and previously published methods. Of note, the affinity of fluorescently labeled probe determined by saturation binding agrees well with the affinity of unlabeled geldanamycin as determined by equilibrium competition binding. All FP assays were repeated in 3 independent experiments. The mean +/- SEM is presented. **c,** Concentration-dependent displacement of fluorescent probe (decrease in millipolarization (mP) units) by test compounds was measured to determine relative Hsp90 binding affinity in cytosolic extracts prepared from human (HEPG2) *vs. C. albicans* (SC5314) cells. EC_{50} values were determined by variable slope 4-parameter curve fitting of duplicate determinations at each concentration tested (R^2>0.95, all curves). The indicated fungal EC_{50}s were normalized by the ratio of geldanamycin EC_{50}s measured in human/fungal lysate to account for disparity in probe affinity between lysates and enable more accurate comparison. All FP assays were repeated in 3 independent experiments. The mean +/- SEM is presented in tabular form. Representative equilibrium binding displacement curves from a single experiment are presented below the table. Source data for the determination of all K_d, K_i and EC_{50} values are provided as a source data file.

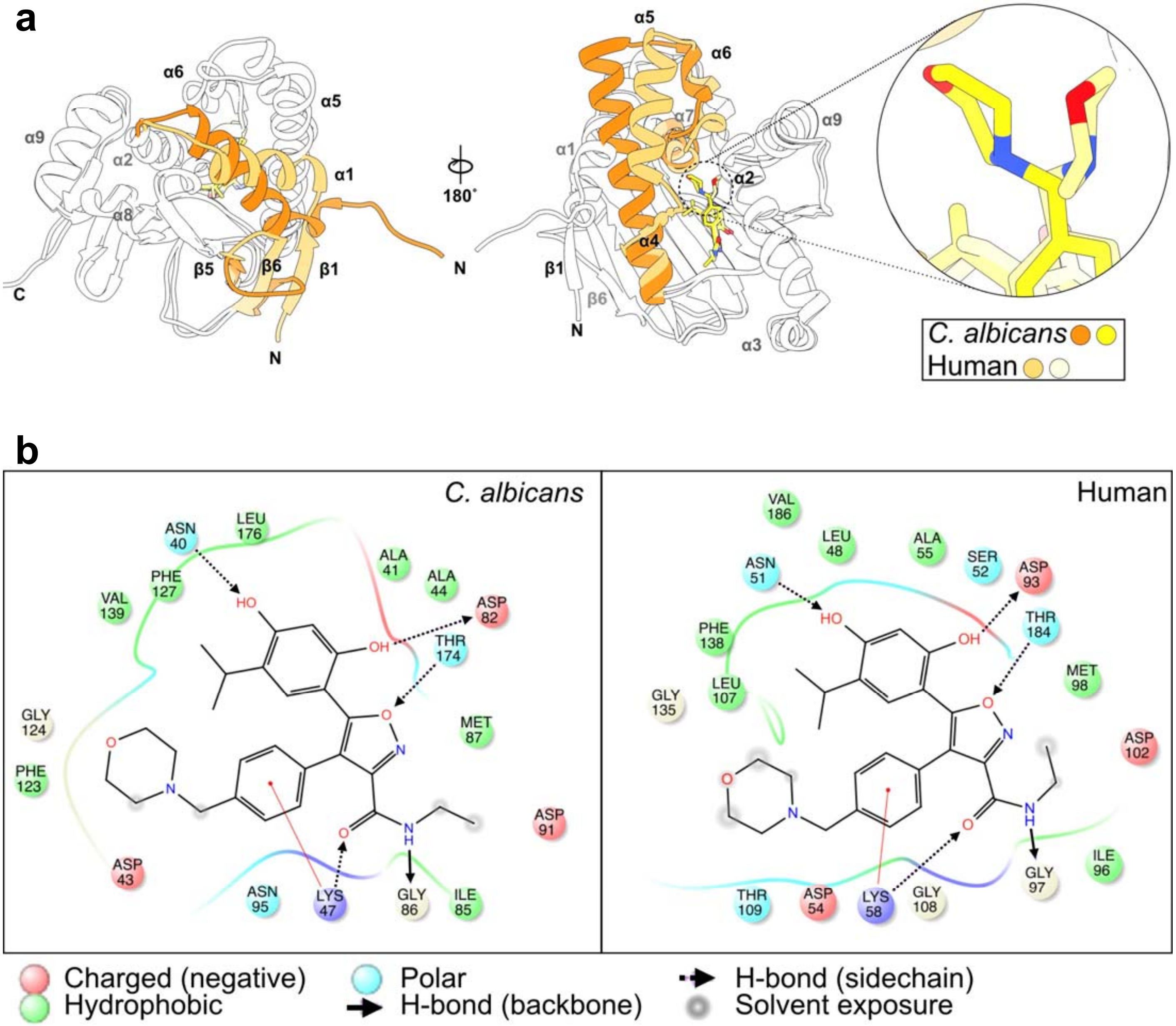

Supplementary Figure 4

Supplementary Figure 4

Structure of *C. albicans* and human NBD in complex with AUY922. a, Overlay of *C. albicans* Hsp90 and human Hsp90α NBD in complex with AUY922. Two orthogonal views of a ribbon representation are shown to indicate structural differences; the colored secondary structure elements highlight the differences between the *C. albicans* (orange/gold) and human (clay/light yellow) structures. A zoomed-in view of the morpholine ring orientation in both complexes is also shown. **b,** Ligand interaction map for the fungal (left) and human (right) complexes, all amino acid residues within 3.5Å of the inhibitor are indicated, and color coded accordingly to their physicochemical properties: charged (red), hydrophobic (green) and polar (cyan). Hydrogen-bond interactions between amino acids within the protein and ligand atoms are indicated by black arrows, the arrow indicates the direction of the interaction, donor to acceptor.

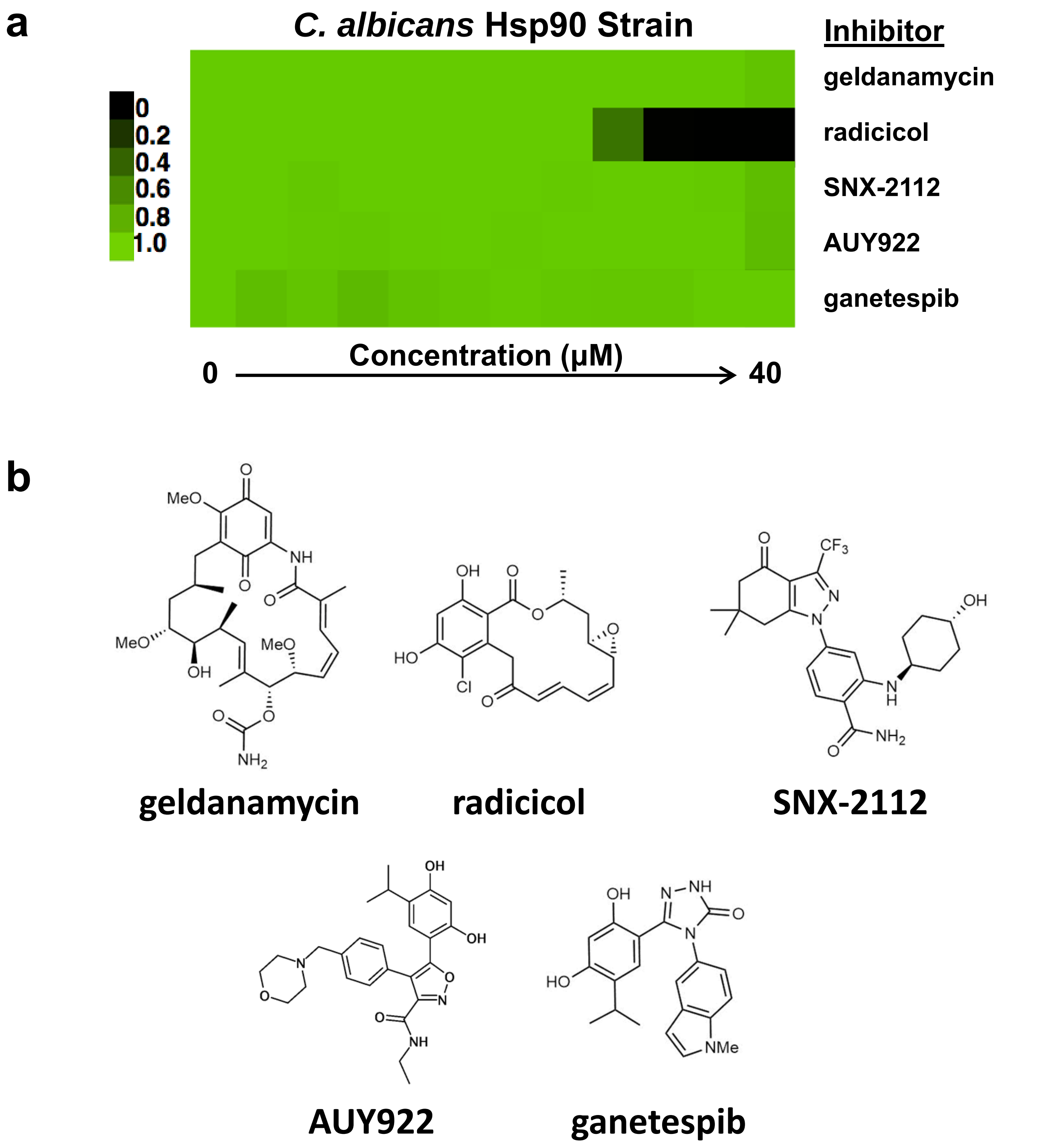

Supplementary Figure 5

Supplementary Figure 5

Activity of known inhibitors against yeast expressing *C. albicans* Hsp90. a, Relative growth inhibition over a 2-fold dilution series of concentrations for each compound is displayed in heat-map format. Each colored box represents the mean of duplicate determinations. Color scale bar for relative growth is provided to the left, green: no inhibition to black: complete inhibition. The experiment was repeated in an independent biological replicate to confirm results. **b,** Chemical structures of inhibitors tested.

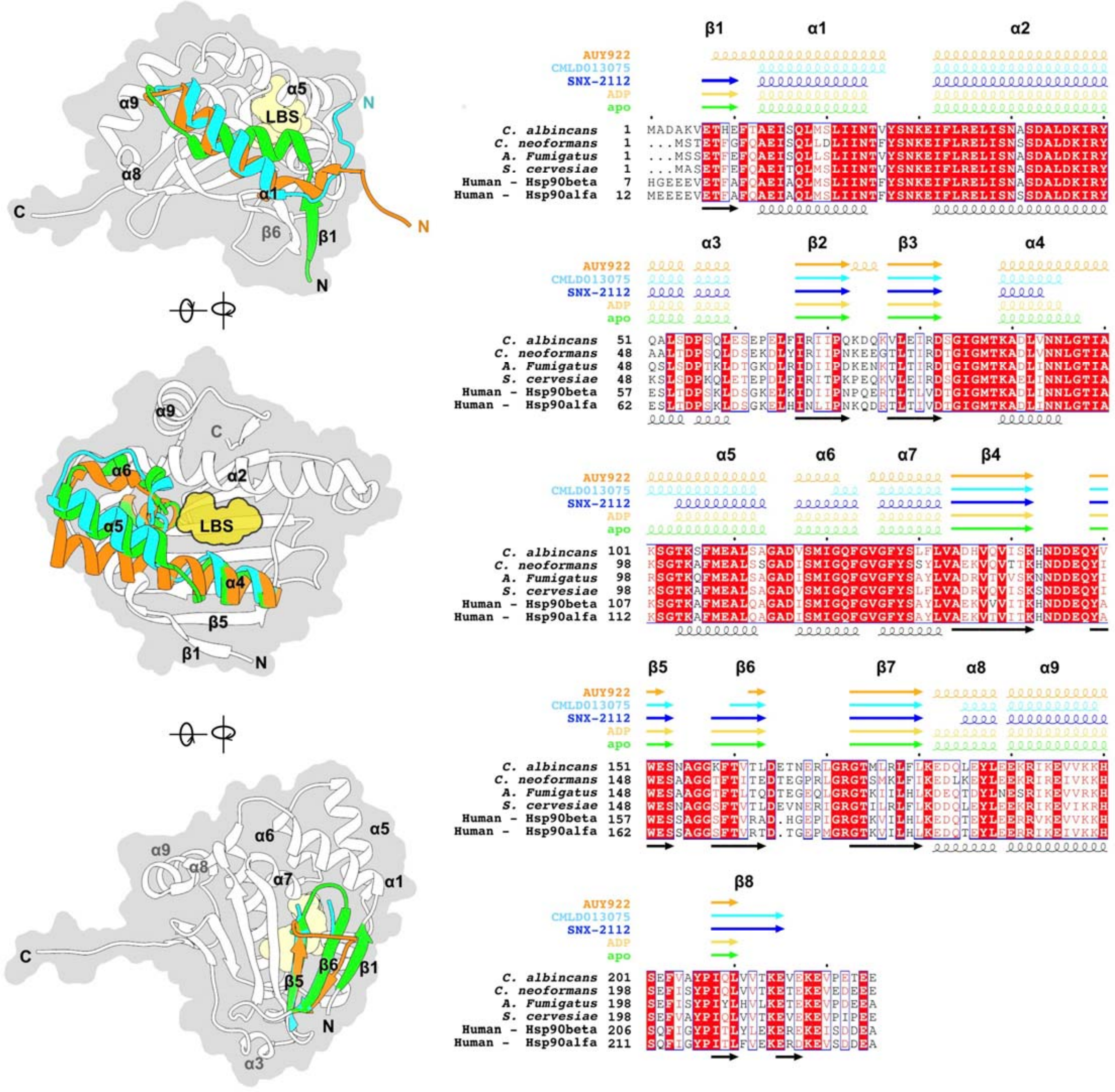

Supplementary Figure 6

Supplementary Figure 6

Ligand-associated rearrangements within the *C. albicans* NBD. *Left column*, Orthogonal views of partial ribbon representations of the Hsp90 NBD when observed in complex with CMLD013075 (aqua) or AUY922 (orange). For each view, a limited region of the protein is colored to highlight conformational changes from the *apo* structure. The conformation of the *apo* (green) state determined from an independent *C. albicans* crystal structure is overlaid, and its overall contour depicted in gray. The green section matches the colored complex structures and white ribbons represent the rest of the *apo* structure. The ligand binding site (LBS) is indicated in yellow. ***Right column,*** Alignment of amino acid sequences making up the Hsp90 NBD in diverse fungi and humans. Color scheme: Red background shading indicates perfectly conserved positions, positions with variation are indicated in red letters when a particular residue is the most common and black letters when the position is more variable. Red shading of white letters indicates completely conserved positions. To highlight the extent of structural variation that occurs in the absence of sequence variability, the secondary structures observed for *C. albicans* NBD in the *apo* state or in complex with various inhibitors is mapped above the sequence alignment. The secondary structures observed for human Hsp90α are indicated in black below the sequence alignment. Gray shading highlights the portion of the alignment that is depicted in drawings to left.

a 3T3 cell cytotoxicity

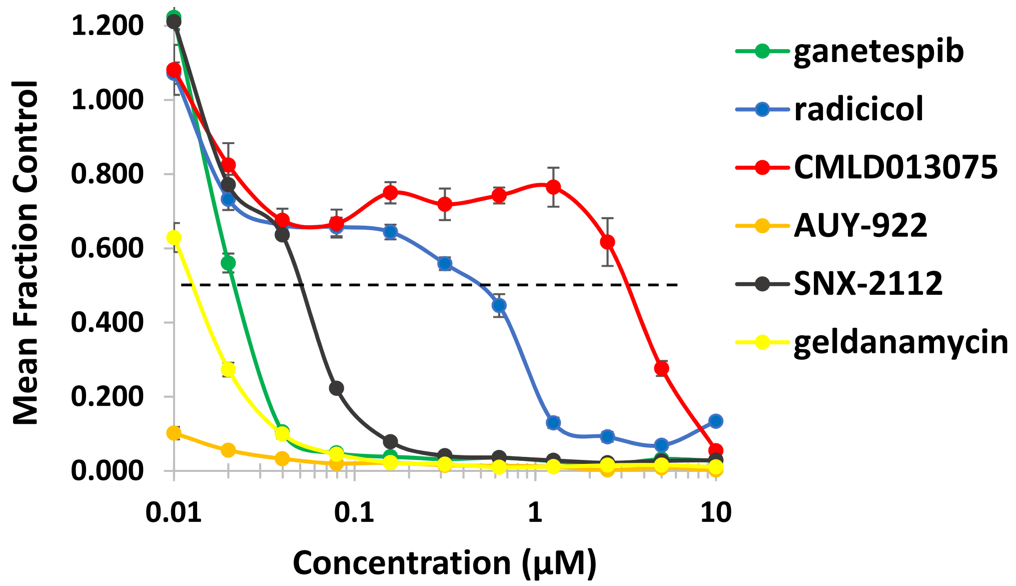

b Relative inhibitor potency in whole cell lysates by FP assay

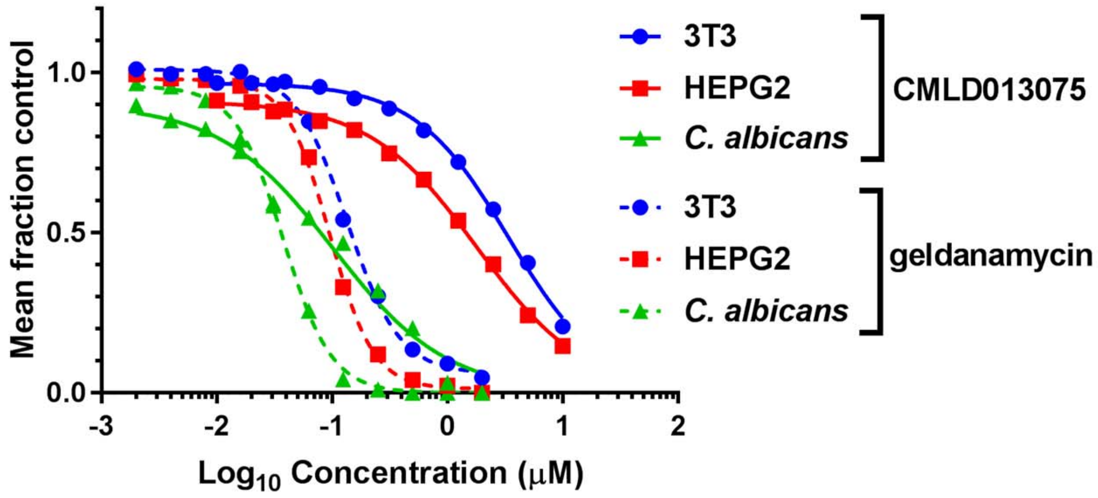

Supplementary Figure 7

Supplementary Figure 7

Fungal selectivity of CMLD013075 reduces mammalian cell toxicity. a, Concentration-dependent inhibition of 3T3 mouse fibroblast growth and survival after 3-day culture with Hsp90 inhibitors. Data points depict the mean of triplicate determinations, Dashed line depicts 50% inhibition of growth/survival. Error bars, SD. The experiment was repeated once with quantitatively similar results. **b,** Relative concentration-dependent displacement of fluorescent probe by CMLD013075 (solid traces) and geldanamycin (dashed traces) is plotted to measure relative Hsp90 binding affinity in cytosolic extracts prepared from non-cancer derived fibroblasts (3T3), cancer-derived liver cells (HEPG2) and fungal (*C. albicans*) cells. In contrast to geldanamycin, CMLD013075 demonstrates higher binding affinity in fungal than mammalian lysates with the lowest binding affinity for the compound seen in non-cancer derived 3T3 cells. Variable slope 4-parameter curve fitting of duplicate determinations at each concentration tested is presented (R^2>0.96, all curves). The experiment was repeated as an independent biological replicate to confirm findings. Source data for the curve fittings are provided as a source data file.

a

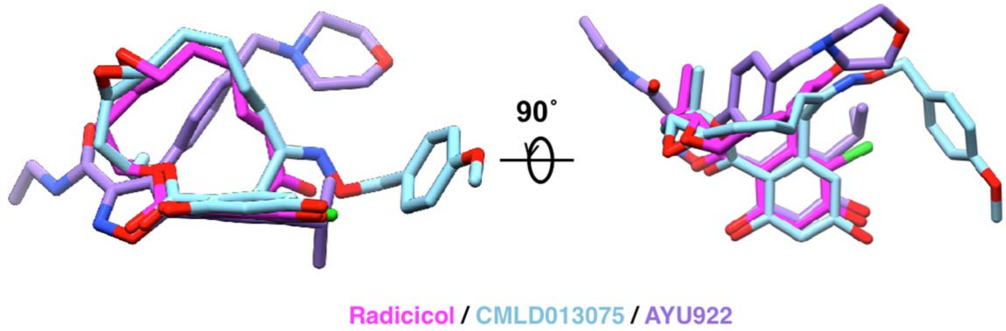

b

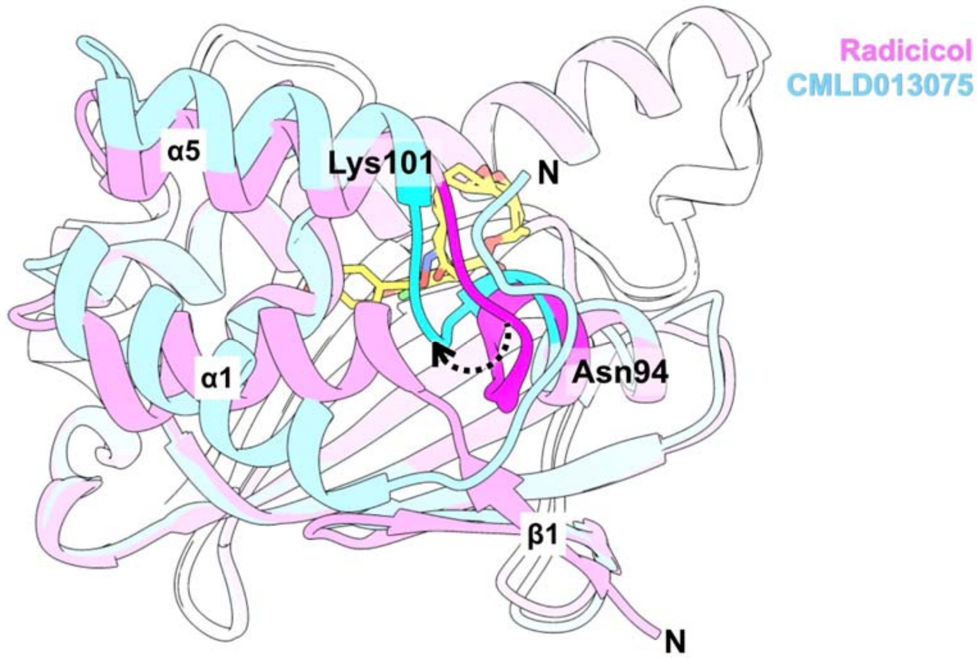

Supplementary Figure 8

Supplementary Figure 8

Partial remodeling of fungal NBD to accommodate binding of CMLD013075. a, Orthogonal views of an overlay of the conformations determined for three different resorcinol-containing compounds when in complex with *C. albicans* Hsp90 NBD. **b**, Ribbon representation of *C. albicans* Hsp90 NBD in complex with radicicol (magenta) and CMLD013075 (cyan) to indicate the bulged-in position of the Asn94-Lys101 region. The remainders of the structures are shown as lighter colored ribbons.

a Checkerboard growth inhibition assay in liquid medium

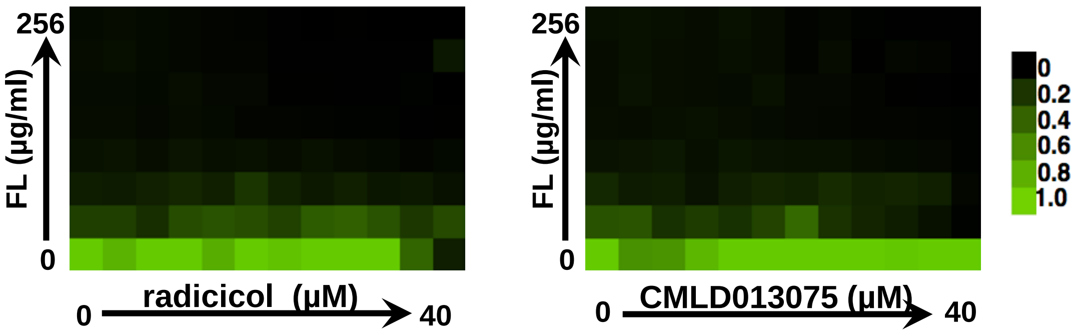

b Survival post exposure (no compounds present)

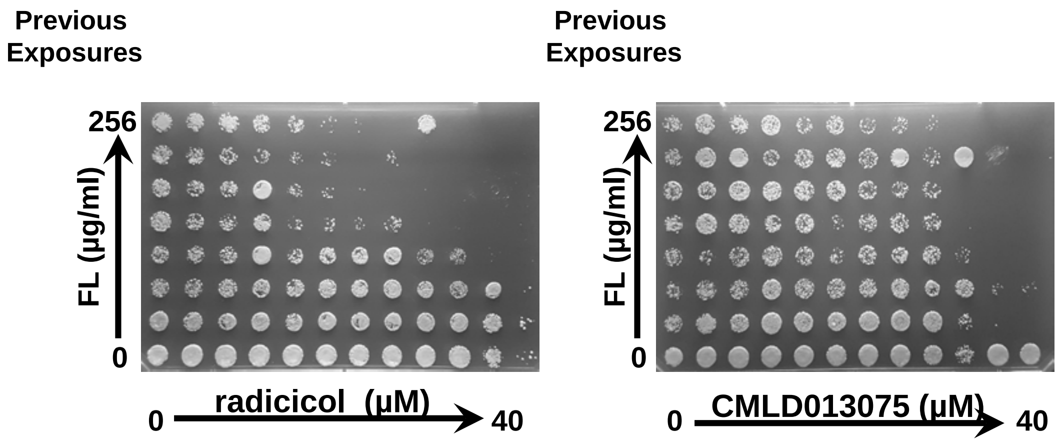

Supplementary Figure 9

Supplementary Figure 9

Combination with FL generates a fungicidal drug combination. **a,** Checkerboard susceptibility assays were performed using a laboratory strain of *C. albicans* (SN95) growing in rich medium (YPD) and incubated at 30 °C for 48 hours. Relative growth inhibition caused by 2-fold serial dilutions of the indicated compounds is displayed in heat-map format. Each colored box represents the mean of duplicate determinations. Color scale for relative growth is provided to right of panel, green: no inhibition to black: complete inhibition. **b,** Following exposure to compounds, aliquots of the culture in each well (2 μL) were spotted onto compound-free YPD agar and plates incubated at 30 °C for an additional 24 hours before macroscopic imaging on a standard flat-bed scanner. The entire experiment consisting of growth in liquid culture followed by spotting onto YPD agar was repeated once.

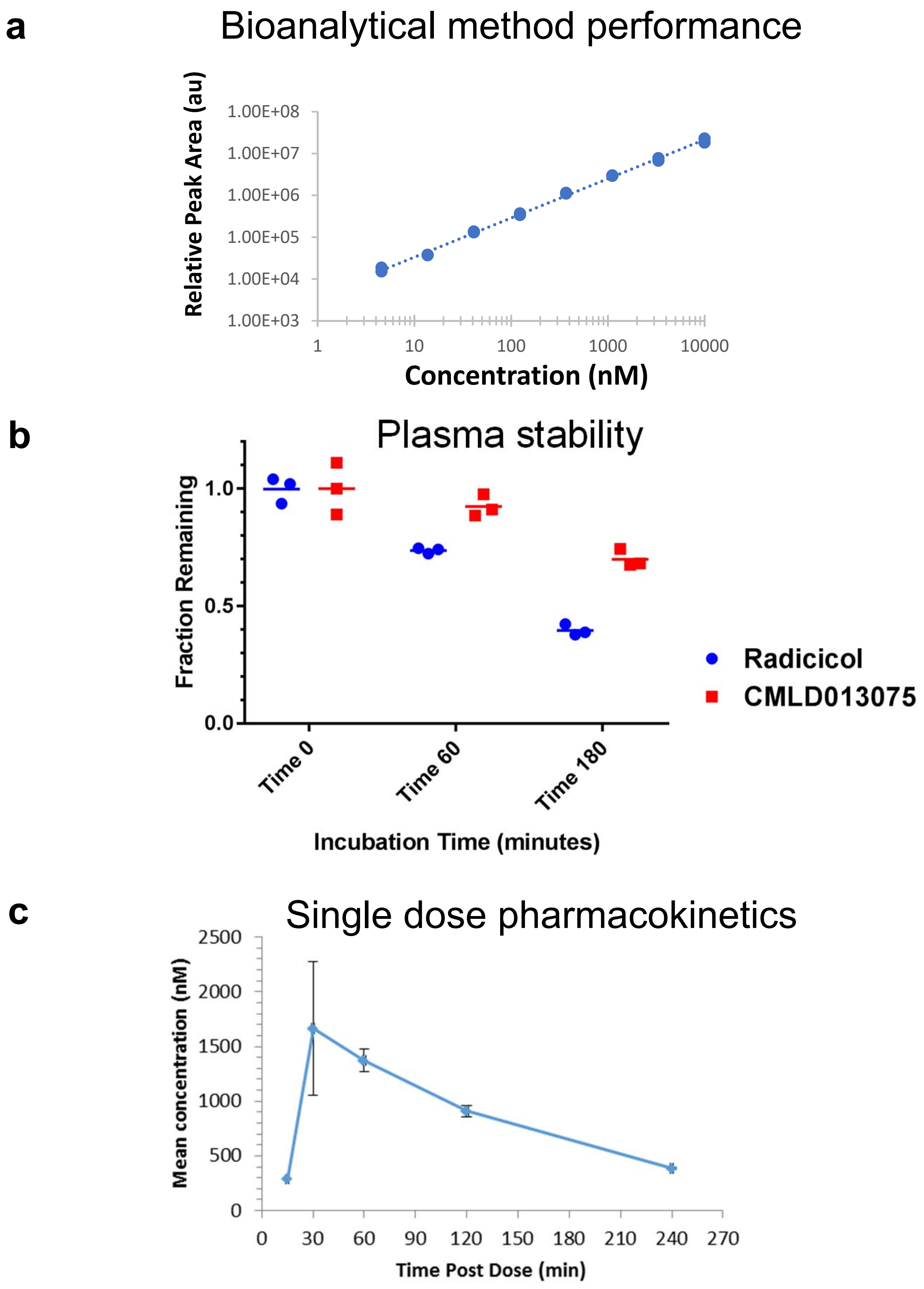

Supplementary Figure 10

Supplementary Figure 10

Pharmacological properties of CMLD013075. a, Serial dilution of compound was performed in mouse plasma. After extraction into cold acetonitrile spiked with an internal standard (imatinib), samples were analyzed by liquid chromatography/ mass spectrometry (LC/MS). Duplicate determinations of relative CMLD013075 peak area normalized to internal standard were made at each concentration and are plotted against concentration. The method demonstrates reliable nanomolar sensitivity and linearity over a 3-log concentration range. Independent standard curves were prepared and measured on 3 independent occasions. **b,** Stability of compounds upon incubation in mouse plasma at 37 ˚C for three hours was measured using the method described in **a**. The results of 3 independent incubations at each time point for each compound is presented. The experiment was performed once. **c,** Single dose plasma pharmacokinetic profiling of CMLD013075 was performed in female 129 sv/jae mice following subcutaneous administration of compound (40 mg/kg) formulated in cremophor vehicle. Plasma was obtained from three mice per time point and compound levels measured using the method described in **a**. Mean plasma concentration +/-SD is plotted. The experiment was performed once.

Quality of ligand electron density maps

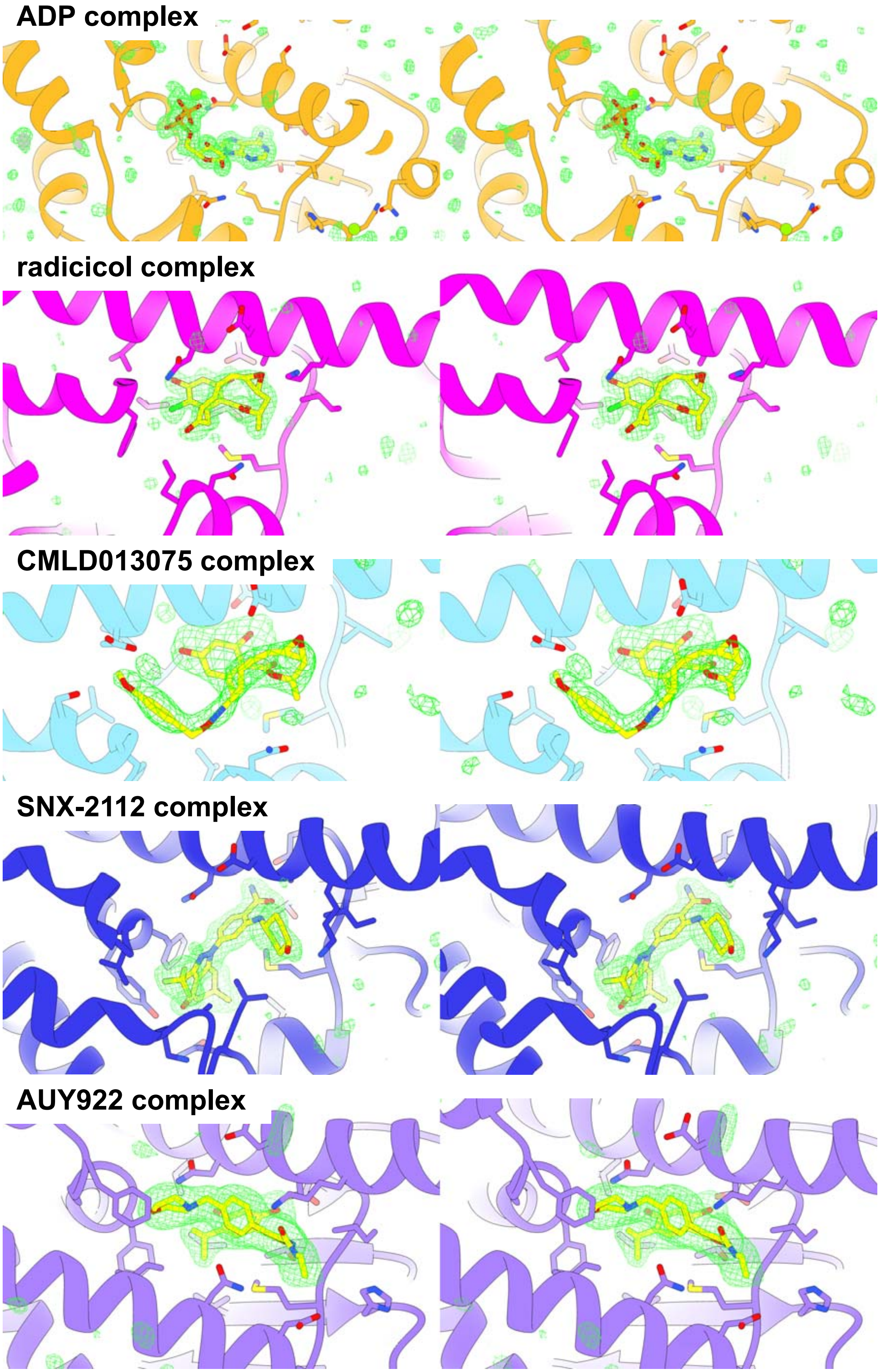

Supplementary Figure 11

Supplementary Figure 11

Quality of ligand electron density observed for co-crystals. Stereo diagram of simple difference simulating- annealing omit maps for complexes with each of the five compounds discussed in the manuscript. The maps are contoured at $+3\delta$ and -3δ colored green and red, respectively.

Supplementary Table 1: Crystallographic data and model refinement statistics

**Ligand**	**None (*apo*)**		**ADP**		**radicicol**		**CMLD013075**		**SNX-2112**		**AUY922**	
PDB id	6CJI		6CJJ		6CJL		6CJP		6CJR		6CJS	
Space group	***P***4(3)22		***P***4(3)22		***P***4(3)		***P***2(1)		***P***4(3)		***I***4(1)22	
Cell dimensions												
a (Å)	73.856		74.088		73.116		40.53		73.63		86.21	
b (Å)	73.856		74.088		73.116		80.11		73.63		86.21	
c (Å)	107.268		107.873		109.499		78.2		109.18		225.85	
**α** (˚)	90		90		90		90		90		90	
**β** (˚)	90		90		90		105.09		90		90	
**γ** (˚)	90		90		90		90		90		90	
Resolution (Å)	1.64		1.74		1.69		2.6		1.79		1.89	
Source	In house		In house		CLS		CLS		APS (23-ID)		APS (23-ID)	
Resolution (Å)	50 - 1.64	1.6 -1.64	50 - 1.74	1.77-1.74	50 - 1.67	1.76-1.67	55 - 2.60	2.74 - 2.6	50 - 1.79	1.86-1.79	43.1-1.89	1.96-1.89
R_{merge}	0.069	1.058	0.076	0.988	0.1	0.517	0.054	0.173	0.078	0.767	0.081	1.85
I/sigI	17.5	3.6	17.1	3.8	14.6	4.7	17	5.9	15.36	1.38	16.9	1.2
Redundancy	14.8	12.7	15.1	12.6	9.9	9.3	4.8	3.9	6.7	4.9	12.9	12.8
Completeness (%)	100	100	100	100	99.9	99.8	96.9	86	99.08	92.81	99.9	99.6
Reflections	37157	1826	31576	1563	66682	9675	14455	1855	53380	4969	34601	3371
**Refinement**												
Resolution (Å)	31.5 - 1.64		33.1 - 1.74		32.7 - 1.69		39.2 - 2.60		27.3 - 1.79		43.1 - 1.90	
No. reflections	37104		31531		63255		14434		53380		34578	
R_{work}	17.3		16.97		14.51		20.3		17.26		16.68	
R_{free}	18.75		19.03		16.53		24.6		22.08		19.34	
Atoms	2084		2043		3810		3207		3678		1936	
Protein		1875		1783		3394		3109		3358		1747
(a.a.)		214		214		426		399		420		218

Ligand/ion	28	41	51	68	37	38
water	181	219	365	30	283	151
B-factors	27.08	28.75	25.72	56.88	35.43	51.77
Protein	25.98	27.84	24.94	57.25	35.2	51.29
Ligand/ion	40.39	30.34	17.39	44.44	33.65	52.22
water	36.39	35.88	34.2	46.8	38.37	57.14
Ramachandran plot (%)						
Favored	94.67	96.54	96.67	96.43	96.6	98.15
Allowed	5.33	3.46	3.33	3.57	3.16	1.85
Outliers	0	0	0	0	0.24	0
RMS bonds	0.009	0.008	0.005	0.003	0.011	0.012
RMS angles	1.041	1.013	1.08	0.741	1.11	1.33

Supplementary Table 2: Superposition r.m.s.d values for apo and ligand-bound structures of *C. albicans* Hsp90

r.m.s.d. (all atoms Å)	**apo**	**ADP**	**RDC**	**CMLD013075**	**AUY922**	**SNX-2112**
apo	**–**					
ADP	**0.75**	**–**				
radicicol	**1.32**	**1.09**	**–**			
CMLD013075	**2.72**	**2.71**	**2.72**	**–**		
AUY922	**3.47**	**3.46**	**3.53**	**2.35**	**–**	
SNX-2112	**1.22**	**1.08**	**1.31**	**2.78**	**3.41**	**–**

Supplementary Table 3: Relative Hsp90 binding affinity of compounds determined in whole cell lysates by FP assay

Compound	SC5314 EC_{50}*	HEPG2 EC_{50}*	Selectivity Ratio**
geldanamycin	17	22	1.0
KF58333	7	10	1.2
**1** (CMLD013075)	505	3000	4.6
**2**	392	353	0.7
**3**	972	2500	2.0
**4**	531	2000	2.9
**5**	1348	3000	1.7
**6**	417	729	1.3
**7**	360	664	1.4
**8**	5	8	1.1

*Concentration (nM) resulting in 50% inhibition of maximal fluorescence polarization signal as determined by 4-parameter curve fit, all fits R^2 >0.99.

**EC_{50} HEPG2/EC_{50} SC5314 normalized to geldanamycin ratio = 1.0

Supplementary Table 4: Strains used in this study.

Strain	Genotype	Source
CaLC79 (CaCi-2)	Prototrophic (Clinical Isolate)	(White 1997)
CaLC155 (SC5314)	Prototrophic	(Odds, Brown et al. 2004)
CaLC239 (SN95)	*arg43Δ/arg4Δ3his13Δ/his1Δ3 URA3/ura3Δ3::imm434 IRO1/iro1Δ3::imm434*	(Noble and Johnson 2005)
CaLC867	*ENO1/ENO1-GFP-NAT*	This study
CaLC922	*ura3 HSP70::HSP70p-lacZ-URA3*	This study
CaLC922	*ura3/ura3 HSP70::HSP70p-lacZ-URA3*	(Shapiro, Uppuluri et al. 2009)
ScLC1963	As ScLC3048 + pAG424-Hsp90α	(Scroggins, Robzyk et al. 2007)
ScLC1964	As ScLC3048 + pAG424-Hsp90β	(Scroggins, Robzyk et al. 2007)
ScLC1965	As ScLC3048 + pAG424-Hsc82	Received from Lindquist Lab
ScLC3048 (4KO)	*can1-100,his3-11,15,leu2-3,112,trp1-1,ura3-1,ade2-1, hsc82::KANmx,hsp82::kanMx pdr1::KANmx, pdr3::KANmx*	Received from Lindquist Lab
ScLC3827	As ScLC3048, + pAG424-CaHsp90 (pLC868)	This study
ScLC5036	As ScLC3048, + pAG424-CaHsp90T12Q (pLC1003)	This study
ScLC5037	As ScLC3048, + pAG424-CaHsp90 L130A F131Y (pLC1004)	This study
ScLC5038	As ScLC3048, + pAG424-CaHsp90 K158S T162R (pLC1004)	This study

Supplementary Table 5: Plasmids used in this study.

Plasmid Name	Description	Source
pLC868	pAG424-CaHsp90	Received from Lindquist Lab
pLC1003	pAG424-CaHsp90^{T12Q}	This study
pLC1004	pAG424-CaHsp90$^{L130A\ F131Y}$	This study
pLC1005	pAG424-CaHsp90$^{K158S\ T162R}$	This study
pET15-MHL	T7-driven expression of recombinant proteins in E. coli	GenBank Accession EF456738

Supplementary Table 6: Oligonucleotides used in this study.

Name	Description	Sequence
**oLC11**	*ScHSC82*-5R	CAGCAGATAGAGCTTCCATG
**oLC321**	*CaHSP90 + 499F*	AACGAAAGATTGGGTCGTGG
**oLC322**	*CaHSP90 + 1033F*	GTGTTTATCACTGATGATGC
**oLC323**	*CaHSP90 + 1599F*	AAAAGCTGCTAGAGAAAAGG
**oLC611**	*CaHSP90+533R*	AATCTCAACATGGTACCACG
**oLC755**	*HSP90+1040-R*	AGTGATAAACACTCTACGGACG
**oLC1869**	*ScURA3*+599R	ATAATCAACCAATCGTAACC
**oLC3437**	*Ca HSP90*+2001F	ATTGATTGCCTTGGGATT
**oLC3723**	pBluescriptKS	TCGAGGTCGACGGTATC
**oLC5120**	*CaHSP90*^{T12Q}-F	GTTGAAACTCACGAATTCCAGGCTGAGATCTCTCAGTTGATG
**oLC5121**	*CaHSP90*^{T12Q}-R	CATCAACTGAGAGATCTCAGCCTGGAATTCGTGAGTTTCAAC
**oLC5122**	*CaHSP90* ^{L130AF131Y}-F	GGTGTTGGTTTCTACTCCGCGTACTTGGTTGCTGATCACGTCCAAG
**oLC5123**	*CaHSP90* ^{L130AF131Y}-R	CTTGGACGTGATCAGCAACCAAGTACGCGGAGTAGAAACCAACACC
**oLC5124**	*CaHSP90* ^{K158ST162R}-F	GGAATCTAACGCTGGTGGTAGCTTCACTGTTCGTTTGGATGAAACTAACG
**oLC5125**	*CaHSP90* ^{K158ST162R}-R	CGTTAGTTTCATCCAAACGAACAGTGAAGCTACCACCAGCGTTAGATTCC

Supplementary Methods

Chemistry: General Methods

^{1}H NMR spectra were recorded at 400 MHz or 500 MHz at ambient temperature with acetone-d_6 as the solvent unless otherwise stated. ^{13}C NMR spectra were recorded at 100 MHz or 125 MHz at ambient temperature with acetone-d_6 as the solvent unless otherwise stated. Chemical shifts are reported in parts per million. Data for ^{1}H NMR are reported as follows: chemical shift, multiplicity (app = apparent, br = broad, s = singlet, d =doublet, t = triplet, q = quartet, m = multiplet) coupling constants and integration. All ^{13}C NMR spectra were recorded with complete proton decoupling. High resolution mass spectra were obtained in the Boston University Chemical Instrumentation Center using a Waters Q-TOF mass spectrometer. Analytical thin layer chromatography was performed using 0.25 mm silica gel 60-F plates. Flash chromatography was performed using 200-400 mesh silica gel (Sorbent Technologies, Inc.) or pre-pack column (SI-HC, puriFlash®) by Interchim puriFlash®450 or Yamazen Smart Flash EPCLC W-Prep2XY system. Yields refer to chromatographically and spectroscopically pure compounds, unless otherwise stated. All reactions were carried out in oven-dried glassware under an argon atmosphere unless otherwise noted. Analytical LC-MS experiments were performed using a Waters Acquity UPLC (Ultra Performance Liquid Chromatography) with a Binary solvent manager, SQ mass spectrometer, Waters 2996 PDA (Photo Diode Array) detector, and Evaporative Light Scattering Detector (ELSD).

Structures and general procedure for the synthesis of oximes 1-8:

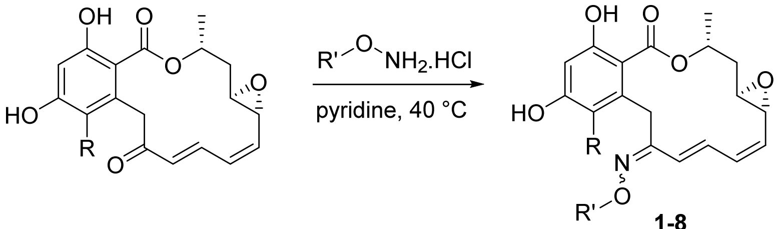

R = Cl; radicicol
R = H; monocillin I

1-8
58-80% yield

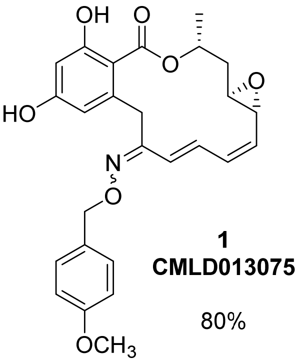

1
CMLD013075
80%

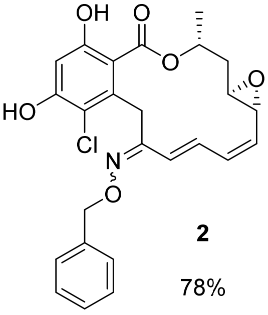

2
78%

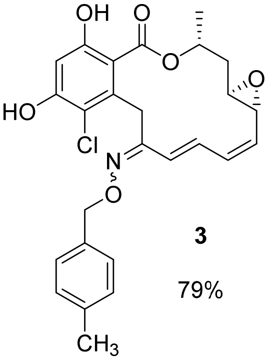

3
79%

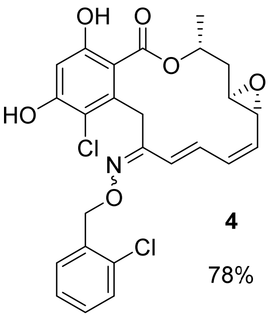

4
78%

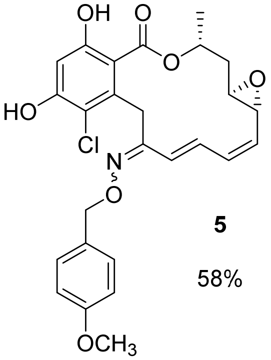

5
58%

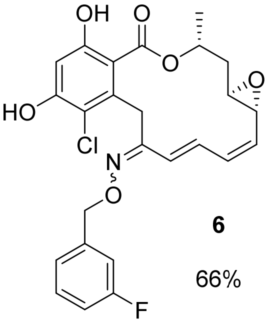

6
66%

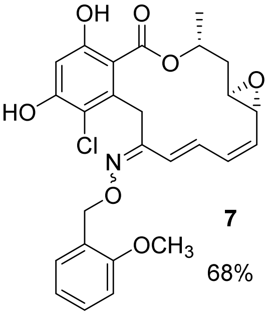

7
68%

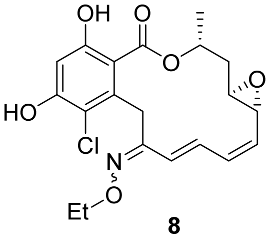

8
62%

To a solution of monocillin I or radicicol in anhydrous pyridine (0.15 M) was added the appropriate hydroxylamine hydrochloride salt (2.5 equivalents). The reaction was then stirred at 40 °C until TLC indicated full consumption of starting material (generally 5-6 hours). The reaction was then allowed to cool to room temperature and diluted with ethyl acetate (10 volume equivalents). Water (1 volume equivalent) is added and the layers are shaken vigorously. The biphasic mixture is then loaded onto a Hydromatrix cartridge of appropriate size to capture the volume of water added and left to sit for 20 minutes. The cartridge was then eluted with approximately 20 volume equivalents

of ethyl acetate. The eluent was concentrated, re-dissolved in a minimal amount of dichloromethane plus 10 mL of heptane and re-concentrated to remove pyridine. This process was repeated three times. The crude residue was purified *via* flash column chromatography using a gradient of acetone in dichloromethane. In the cases where this purification was insufficient, a second column was run using a gradient of ethyl acetate in hexanes to remove remaining impurities.

Full characterization data for oximes 1-8:

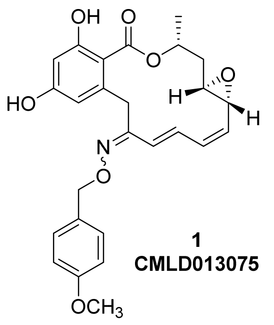

1
CMLD013075

1a*R*,2*Z*,4*E*,14*R*,15a*S*)-9,11-dihydroxy-6-(((4-methoxybenzyl)oxy)imino)-14-methyl-1a,6,7,14,15,15a-hexahydro-12*H*-benzo[*c*]oxireno[2,3-*k*][1]oxacyclotetradecin-12-one (1, CMLD013075). Obtained from reaction of monocillin I and 1-[(ammoniooxy)methyl]-3-fluorobenzenechloride according to the General Procedure (80% yield, 3:1 ratio of *E*:*Z* isomers) after one column, 1% to 5% acetone in dichloromethane.

^{1}H NMR (*E*)-isomer: (400 MHz, Acetone-d_6) δ 10.96 (s, 1H), 9.16 (s, 1H), 7.47 – 7.33 (m, 3H), 7.01 – 6.88 (m, 2H), 6.82 (d, *J* = 15.9 Hz, 1H), 6.49 (dd, *J* = 2.5, 1.0 Hz, 1H), 6.34 – 6.19 (m, 2H), 5.67 (dd, *J* = 10.4, 3.2 Hz, 1H), 5.62 – 5.46 (m, 1H), 5.22 – 5.08 (m, 2H), 4.72 (d, *J* = 15.2 Hz, 1H), 3.80 (s, 3H), 3.46 (d, *J* = 15.2 Hz, 1H), 3.23 (dt, *J* = 3.6, 1.9 Hz, 1H), 3.09 (dt, *J* = 9.3, 2.6 Hz, 1H), 2.44 (dq, *J* = 14.8, 2.7, 2.0 Hz, 1H), 1.80 (ddd, *J* = 14.5, 11.0, 3.9 Hz, 1H), 1.63 (d, *J* = 6.9 Hz, 3H).

^{1}H NMR (*Z*)-isomer: (400 MHz, Acetone-d_6) δ 10.96 (s, 1H), 9.16 (s, 1H), 7.52 – 7.22 (m, 2H), 7.02 – 6.88 (m, 2H), 6.39 – 6.05 (m, 2H), 5.54 (tq, *J* = 6.7, 3.3 Hz, 2H), 5.16 (dd, *J* = 11.3, 2.2 Hz, 2H), 4.40 (d, *J* = 14.7 Hz, 1H), 4.32 (s, 1H), 3.79 (s, 3H), 3.23 (dt, *J* = 3.7, 1.8 Hz, 1H), 3.08 (ddt, *J* = 7.2, 4.6, 2.6 Hz, 1H), 2.44 (dq, *J* = 14.8, 2.7, 2.1 Hz, 1H), 1.95 – 1.71 (m, 1H), 1.61 (d, *J* = 7.9 Hz, 3H).

^{13}C NMR (mixture of *E* and *Z* isomers): (101 MHz, Acetone-d_6) δ 170.6, 165.0, 162.8, 160.5, 160.4, 159.2, 156.0, 143.5, 142.2, 134.4, 133.4, 131.9, 131.6, 130.9, 130.8, 130.7, 130.4, 129.8, 120.8, 114.5, 110.2, 110.0, 106.4, 102.5, 102.3, 76.7, 76.6, 71.7, 56.0, 55.9, 55.9, 55.5, 37.1, 37.1, 34.5, 18.7. UPLC-MS calc *m/z* $[C_{26}H_{27}O_7N+H]^+$: 466.1866, found: 466.147

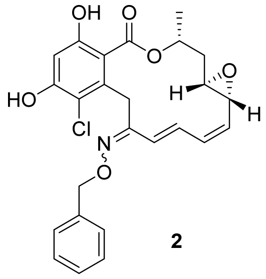

2

(1a*R*,2*Z*,4*E*,14*R*,15a*S*)-6-((benzyloxy)imino)-8-chloro-9,11-dihydroxy-14-methyl-1a,6,7,14,15,15a-hexahydro-12*H*-benzo[*c*]oxireno[2,3-*k*][1]oxacyclotetradecin-12-one (2). Obtained from reaction of radicicol and *O*-benzylhydroxylamine hydrochloride according to the General Procedure (78% yield, 1:2.2 ratio of *E*:*Z* isomers) after one column, 1% to 4% acetone in dichloromethane. ^1H NMR and ^{13}C NMR resonances matched previously reported chemical shifts (Shinonaga, Noguchi et al. 2009).

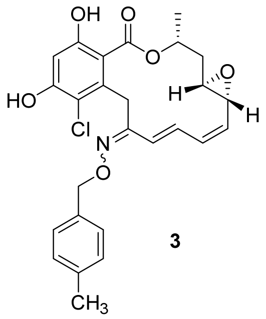

3

(1a*R*,2*Z*,4*E*,14*R*,15a*S*)-8-chloro-9,11-dihydroxy-14-methyl-6-(((4-methylbenzyl)oxy)imino)-1a,6,7,14,15,15a-hexahydro-12*H*-benzo[*c*]oxireno[2,3-*k*][1]oxacyclotetradecin-12-one (3). Obtained from reaction of radicicol and 1-[(ammoniooxy)methyl]-4-methylbenzene chloride according to the General Procedure (79% yield, 1:1.6 ratio of *E*:*Z* isomers) after one column, 1% to 3% acetone in dichloromethane.

^1H NMR (*E*)-isomer: (500 MHz, Acetone-d_6) δ 9.49 (s, 1H), 7.34 (d, *J* = 7.8 Hz, 2H), 7.19 – 7.17 (m, 2H), 7.15 – 7.09 (m, 1H), 6.77 (d, *J* = 16.1 Hz, 0H), 6.54 (s, 1H), 6.25 – 6.09 (m, 2H), 5.48 (dd, *J* = 10.5, 3.4 Hz, 1H), 5.36 (qt, *J* = 6.7, 3.8 Hz, 1H), 5.18 (s, 2H), 4.65 (d, *J* = 16.3 Hz, 1H), 3.75 (d, *J* = 16.4 Hz, 1H), 3.24 – 3.14 (m, 1H), 2.95 (ddd, *J* = 9.4, 3.4, 2.2 Hz, 1H), 2.49 – 2.39 (m, 1H), 2.32 (s, 3H), 1.70 – 1.59 (m, 1H), 1.54 (d, *J* = 6.6 Hz, 3H).

^1H NMR (*Z*)-isomer: (500 MHz, Acetone-d_6) δ 9.49 (s, 1H), 7.30 (d, *J* = 7.8 Hz, 2H), 7.27 – 7.21 (m, 2H), 7.20 – 7.15 (m, 2H), 6.77 (d, *J* = 16.1 Hz, 1H), 6.54 (d, *J* = 1.9 Hz, 1H), 6.25 – 6.09 (m,

1H), 5.58 (dd, *J* = 10.6, 3.7 Hz, 1H), 5.36 (qt, *J* = 6.7, 3.8 Hz, 1H), 5.18 – 5.08 (m, 2H), 4.12 (d, *J* = 16.0 Hz, 1H), 4.04 – 3.96 (m, 1H), 3.30 (dt, *J* = 3.7, 1.9 Hz, 0H), 3.02 (ddd, *J* = 8.7, 3.7, 2.2 Hz, 1H), 2.49 – 2.39 (m, 1H), 2.32 (s, 3H), 1.66 (ddd, *J* = 15.0, 8.8, 4.2 Hz, 1H), 1.56 (d, *J* = 6.6 Hz, 3H).

^{13}C NMR (mixture of *E* and *Z* isomers): (101 MHz, Acetone-d_6) δ 168.3, 168.1, 158.4, 158.1, 157.1, 157.0, 155.2, 154.2, 138.2, 138.0, 137.0, 136.6, 136.3, 135.8, 132.9, 132.8, 132.2, 132.0, 131.4, 130.5, 130.1, 129.7, 129.6, 129.5, 129.3, 129.0, 127.4, 121.8, 115.2, 114.0, 113.8, 103.5, 103.3, 76.9, 76.6, 71.9, 56.1, 55.9, 55.7, 37.4, 35.8, 28.7, 21.2, 18.7, 18.4.

HRMS calc *m/z* $[C_{26}H_{26}O_6NCl+H]^+$: 484.1527, found 484.1510.

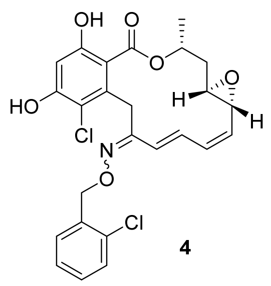

4

(1a*R*,2*Z*,4*E*,14*R*,15a*S*)-8-chloro-6-(((2-chlorobenzyl)oxy)imino)-9,11-dihydroxy-14-methyl-1a,6,7,14,15,15a-hexahydro-12*H*-benzo[*c*]oxireno[2,3-*k*][1]oxacyclotetradecin-12-one (4). Obtained from reaction of radicicol and 1-[(ammoniooxy)methyl]-4-methylbenzene chloride according to the General Procedure (78% yield, 1:4.3 ratio of E:Z isomers) after one column, 1% to 3% acetone in dichloromethane.

^{1}H NMR (*E*)-isomer: (400 MHz, Acetone-d_6) δ 7.60 (dd, *J* = 5.7, 3.7 Hz, 1H), 7.46 – 7.40 (m, 1H), 7.35 (td, *J* = 6.1, 3.4 Hz, 2H), 7.22 – 7.08 (m, 1H), 6.55 (s, 1H), 6.29 – 6.06 (m, 2H), 5.49 (dd, *J* = 10.6, 3.4 Hz, 1H), 5.40 – 5.32 (m, 3H), 4.68 (d, *J* = 16.4 Hz, 1H), 3.80 (d, *J* = 16.5 Hz, 1H), 3.25 – 3.19 (m, 1H), 2.98 – 2.92 (m, 1H), 2.45 (ddt, *J* = 13.7, 9.8, 3.5 Hz, 1H), 1.65 (dq, *J* = 13.7, 4.6 Hz, 1H), 1.56 (d, *J* = 7.3 Hz, 3H).

^{1}H NMR (*Z*)-isomer: (400 MHz, Acetone-d_6) δ 7.54 (dd, *J* = 5.8, 3.6 Hz, 1H), 7.48 – 7.41 (m, 1H), 7.35 (td, *J* = 6.1, 3.4 Hz, 2H), 7.29 (dd, *J* = 16.1, 11.2 Hz, 1H), 6.84 (d, *J* = 16.2 Hz, 1H), 6.55 (s, 1H), 6.32 – 6.08 (m, 1H), 5.61 (dd, *J* = 10.5, 3.7 Hz, 1H), 5.40 – 5.35 (m, 1H), 5.29 (d, *J* = 1.8 Hz, 2H), 4.13 (d, *J* = 16.1 Hz, 1H), 3.99 (d, *J* = 16.1 Hz, 1H), 3.31 (d, *J* = 3.6 Hz, 1H), 3.04

(dt, J = 8.5, 3.2 Hz, 1H), 2.45 (ddt, J = 13.7, 9.8, 3.5 Hz, 1H), 1.66 (ddt, J = 13.7, 8.9, 4.4 Hz, 1H), 1.57 (d, J = 6.8 Hz, 3H).

^{13}C NMR (mixture of *E* and *Z* isomers): (101 MHz, Acetone-d_6) δ 168.3, 168.1, 158.6, 158.2, 157.1, 157.1, 156.1, 155.1, 137.0, 136.9, 136.5, 136.3, 134.0, 133.5, 133.3, 133.2, 132.1, 131.9, 131.8, 131.3, 130.9, 130.8, 130.3, 130.1, 130.0, 129.7, 127.8, 127.7, 121.6, 115.2, 114.3, 113.9, 113.7, 103.5, 103.4, 74.0, 73.7, 73.6, 73.6, 72.1, 71.9, 56.1, 55.9, 55.6, 37.4, 35.7, 28.9, 18.7, 18.4.

HRMS calc *m/z* [$C_{25}H_{23}O_6NCl_2$+H]$^+$: 504.0981, found 504.0996.

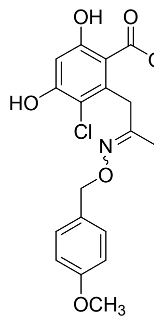

5

(1a*R*,2*Z*,4*E*,14*R*,15a*S*)-8-chloro-9,11-dihydroxy-6-(((4-methoxybenzyl)oxy)imino)-14-methyl-1a,6,7,14,15,15a-hexahydro-12*H*-benzo[*c*]oxireno[2,3-*k*][1]oxacyclotetradecin-12-one (5).

Obtained from reaction of radicicol and 1-[(ammoniooxy)methyl]-3-fluorobenzenechloride according to the General Procedure (58% yield, 1:2.4 ratio of *E*:*Z* isomers) after one column, 2% acetone in dichloromethane.

^{1}H NMR (*E*)-isomer: (400 MHz, Acetone-d_6) δ 9.51 (s, 1H), 7.44 – 7.30 (m, 2H), 7.15 (dd, J = 16.2, 11.1 Hz, 1H), 6.98 – 6.86 (m, 2H), 6.54 (s, 1H), 6.24 – 6.04 (m, 2H), 5.52 – 5.42 (m, 1H), 5.41 – 5.30 (m, 1H), 5.15 (s, 2H), 4.63 (d, J = 16.3 Hz, 1H), 3.80 (d, J = 1.5 Hz, 3H), 3.72 (d, J = 16.4 Hz, 1H), 3.23 (d, J = 3.0 Hz, 1H), 2.98 – 2.91 (m, 1H), 2.45 (dq, J = 12.6, 4.2 Hz, 1H), 1.70 – 1.60 (m, 1H), 1.54 (d, J = 8.8 Hz, 3H).

^{1}H NMR (*Z*)-isomer: (400 MHz, Acetone-d_6) δ 9.51 (s, 1H), 7.46 – 7.28 (m, 2H), 7.24 (dd, J = 16.2, 11.2 Hz, 1H), 6.97 – 6.83 (m, 2H), 6.75 (d, J = 16.2 Hz, 1H), 6.54 (s, 1H), 6.32 – 6.08 (m, 1H), 5.58 (dd, J = 10.7, 3.6 Hz, 1H), 5.36 (tt, J = 7.1, 3.8 Hz, 1H), 5.15 – 5.03 (m, 2H), 4.11 (d, J = 16.0 Hz, 1H), 4.00 (d, J = 16.4 Hz, 1H), 3.80 (s, 3H), 3.32 – 3.26 (m, 1H), 3.07 – 2.99 (m, 1H), 2.45 (dq, J = 12.6, 4.2 Hz, 1H), 1.71 – 1.60 (m, 1H), 1.56 (d, J = 6.1 Hz, 3H).

^{13}C NMR (mixture of *E* and *Z* isomers): (101 MHz, Acetone-d_6) δ 168.3, 168.1, 160.4, 160.3, 158.4, 158.1, 157.0, 157.0, 155.1, 154.1, 137.0, 136.6, 132.8, 132.8, 132.2, 132.1, 131.4, 131.2, 130.9, 130.7, 130.5, 130.2, 121.9, 115.2, 114.4, 114.4, 114.0, 113.9, 103.5, 103.3, 76.8, 76.4, 72.0, 71.9, 71.9, 56.1, 55.9, 55.7, 55.5, 55.5, 37.4, 35.8, 28.7, 18.7, 18.4.

HRMS calc *m/z* $[C_{26}H_{26}O_7NCl+H]^+$: 500.1476, found 500.1490.

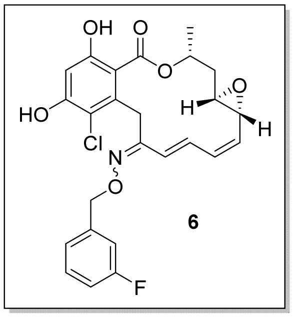

(1a*R*,2*Z*,4*E*,14*R*,15a*S*)-8-chloro-6-(((3-fluorobenzyl)oxy)imino)-9,11-dihydroxy-14-methyl-1a,6,7,14,15,15a-hexahydro-12*H*-benzo[c]oxireno[2,3-*k*][1]oxacyclotetradecin-12-one (6). Obtained from reaction of radicicol and 1-[(ammoniooxy)methyl]-3-fluorobenzenechloride according to the General Procedure (66% yield, 1:2.9 ratio of *E*:*Z* isomers) after one column, 1% to 3% acetone in dichloromethane.

^1H NMR (*E*)-isomer: (400 MHz, Acetone-d_6) δ 7.40 (td, *J* = 7.7, 6.0 Hz, 1H), 7.33 – 7.14 (m, 3H), 7.11 – 7.01 (m, 1H), 6.54 (s, 1H), 6.32 – 6.09 (m, 2H), 5.49 (dd, *J* = 10.5, 3.3 Hz, 1H), 5.36 (td, *J* = 6.9, 3.5 Hz, 1H), 5.25 (s, 2H), 4.67 (d, *J* = 16.4 Hz, 1H), 3.79 (d, *J* = 16.4 Hz, 1H), 3.22 (d, *J* = 3.8 Hz, 1H), 2.99 – 2.93 (m, 1H), 2.46 (ddt, *J* = 14.4, 10.7, 3.5 Hz, 1H), 1.65 (ddd, *J* = 17.3, 8.3, 4.1 Hz, 1H), 1.55 (d, *J* = 7.6 Hz, 3H).

^1H NMR (*Z*)-isomer: (400 MHz, Acetone-d_6) δ 7.46 – 7.35 (m, 1H), 7.32 – 7.15 (m, 3H), 7.12 – 6.96 (m, 1H), 6.82 (d, *J* = 16.2 Hz, 1H), 6.54 (s, 1H), 6.31 – 6.08 (m, 1H), 5.61 (dd, *J* = 10.7, 3.8 Hz, 1H), 5.36 (td, *J* = 6.9, 3.5 Hz, 1H), 5.20 (s, 2H), 4.12 (d, *J* = 16.1 Hz, 1H), 3.99 (d, *J* = 16.1 Hz, 1H), 3.30 (d, *J* = 3.6 Hz, 1H), 3.08 – 3.00 (m, 1H), 2.46 (ddt, *J* = 14.4, 10.7, 3.5 Hz, 1H), 1.65 (ddd, *J* = 17.3, 8.3, 4.1 Hz, 1H), 1.57 (d, *J* = 6.9 Hz, 3H).

^{19}F NMR (mixture of *E* and *Z* isomers): (376 MHz, Acetone-d_6) δ -114.94 – -115.07 (m).

^{13}C NMR (mixture of *E* and *Z* isomers): (101 MHz, Acetone-d_6) δ 168.3, 168.1, 164.8, 162.4, 158.5, 158.2, 157.1, 157.1, 155.9, 154.9, 142.7, 142.6, 142.0, 141.9, 136.9, 136.5, 133.3, 133.2, 132.1, 132.0, 131.8, 131.0, 130.9, 130.9, 130.8, 129.8, 124.7, 124.7, 124.4, 124.4, 121.7, 115.6,

115.4, 115.3, 115.3, 115.1, 115.1, 114.9, 114.2, 114.0, 113.8, 103.5, 103.4, 103.4, 76.0, 75.6, 75.6, 72.1, 71.9, 70.2, 56.1, 55.9, 55.7, 37.4, 35.7, 28.8, 18.7, 18.4.

HRMS calc *m/z* $[C_{25}H_{23}O_6NClF+H]^+$: 488.1276, found 488.1289.

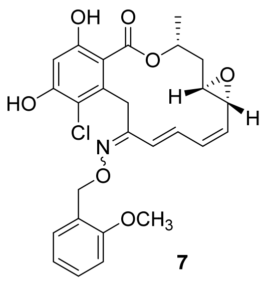

7

(1a*R*,2*Z*,4*E*,14*R*,15a*S*)-8-chloro-9,11-dihydroxy-6-(((2-methoxybenzyl)oxy)imino)-14-methyl-1a,6,7,14,15,15a-hexahydro-12*H*-benzo[*c*]oxireno[2,3-*k*][1]oxacyclotetradecin-12-one (7). Obtained from reaction of radicicol and 1-[(ammoniooxy)methyl]-2-methoxybenzene chloride according to the General Procedure (68% yield, 1:3 ratio of *E*:*Z* isomers) after one column, 1% to 3% acetone in dichloromethane.

^{1}H NMR (*E*)-isomer: (400 MHz, Acetone-d_6) δ 7.47 – 7.34 (m, 1H), 7.32 – 7.19 (m, 2H), 7.15 (dd, *J* = 16.2, 11.1 Hz, 0H), 7.06 – 6.88 (m, 2H), 6.54 (d, *J* = 2.4 Hz, 1H), 6.27 – 6.06 (m, 2H), 5.48 (dd, *J* = 10.3, 3.4 Hz, 1H), 5.36 (dt, *J* = 7.1, 3.8 Hz, 1H), 5.25 (d, *J* = 9.8 Hz, 2H), 4.67 (d, *J* = 16.4 Hz, 1H), 3.86 (s, 3H), 3.76 (d, *J* = 16.2 Hz, 1H), 3.23 (s, 1H), 2.96 (d, *J* = 9.0 Hz, 1H), 2.52 – 2.37 (m, 1H), 1.72 – 1.62 (m, 1H), 1.56 (d, *J* = 7.3 Hz, 3H).

^{1}H NMR (*Z*)-isomer: (400 MHz, Acetone-d_6) δ 7.38 (dd, *J* = 7.6, 1.6 Hz, 1H), 7.31 – 7.19 (m, 2H), 7.04 – 6.88 (m, 2H), 6.82 (d, *J* = 16.2 Hz, 1H), 6.54 (d, *J* = 2.4 Hz, 1H), 6.27 – 6.07 (m, 1H), 5.65 – 5.55 (m, 1H), 5.36 (dt, *J* = 7.1, 3.8 Hz, 1H), 5.21 (s, 2H), 4.12 (d, *J* = 16.0 Hz, 1H), 3.99 (d, *J* = 16.1 Hz, 1H), 3.86 (s, 3H), 3.31 (d, *J* = 3.4 Hz, 1H), 3.10 – 2.99 (m, 1H), 2.51 – 2.38 (m, 1H), 1.72 – 1.60 (m, 1H), 1.57 (d, *J* = 6.8 Hz, 3H).

^{13}C NMR (mixture of *E* and *Z* isomers): (101 MHz, Acetone-d_6) δ 168.4, 168.1, 158.5, 158.3, 158.1, 158.0, 157.1, 157.0, 155.2, 154.3, 137.1, 136.6, 132.9, 132.2, 132.1, 131.3, 130.5, 130.4, 130.1, 130.0, 129.9, 129.6, 127.4, 126.7, 121.9, 121.0, 120.9, 115.2, 114.3, 114.1, 113.8, 111.3, 111.2, 103.5, 103.5, 103.3, 72.0, 72.0, 71.9, 71.6, 71.6, 56.1, 56.0, 55.8, 55.7, 55.7, 55.7, 37.4, 35.8, 28.8, 18.7, 18.4.

HRMS calc *m/z* $[C_{26}H_{26}O_7NCl+H]^+$: 500.1476, found 500.1498.

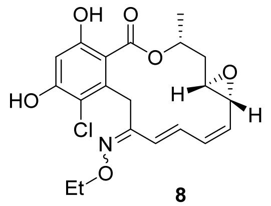
8

(1a*R*,2*Z*,4*E*,14*R*,15a*S*)-8-chloro-6-(ethoxyimino)-9,11-dihydroxy-14-methyl-1a,6,7,14,15,15a-hexahydro-12*H*-benzo[*c*]oxireno[2,3-*k*][1]oxacyclotetradecin-12-one (8). Obtained from reaction of radicicol and *O*-ethylhydroxylamine hydrochloride according to the General Procedure (62% yield, 1:2.8 ratio of *E*:*Z* isomers) after two columns (1% to 5% acetone in dichloromethane, followed by 20% to 50% ethyl acetate in hexanes).

^{1}H NMR (*E*)-isomer: (400 MHz, Acetone-d_6) δ 9.53 (s, 1H), 7.14 (dd, *J* = 16.1, 11.1 Hz, 1H), 6.55 (s, 1H), 6.29 – 6.07 (m, 2H), 5.47 (dd, *J* = 10.5, 3.5 Hz, 1H), 5.36 (dq, *J* = 7.2, 4.2, 3.6 Hz, 1H), 4.62 (d, *J* = 16.3 Hz, 1H), 4.22 (q, *J* = 7.1 Hz, 2H), 3.68 (d, *J* = 16.3 Hz, 1H), 3.26 – 3.19 (m, 1H), 3.06 – 2.98 (m, 1H), 2.56 – 2.32 (m, 1H), 1.77 – 1.59 (m, 1H), 1.55 (d, *J* = 6.2 Hz, 3H), 1.36 – 1.10 (m, 3H).

^{1}H NMR (*Z*)-isomer: (400 MHz, Acetone-d_6) δ 9.53 (s, 1H), 7.24 (dd, *J* = 16.2, 11.3 Hz, 1H), 6.73 (d, *J* = 16.2 Hz, 1H), 6.55 (s, 1H), 6.29 – 6.07 (m, 1H), 5.58 (dd, *J* = 10.5, 3.7 Hz, 1H), 5.36 (dq, *J* = 7.2, 4.2, 3.6 Hz, 1H), 4.15 (qd, *J* = 7.0, 1.1 Hz, 2H), 4.09 (d, *J* = 16.0 Hz, 1H), 4.01 (d, *J* = 16.1 Hz, 1H), 3.31 (dt, *J* = 3.9, 1.9 Hz, 1H), 3.06 – 2.98 (m, 1H), 2.56 – 2.32 (m, 1H), 1.77 – 1.59 (m, 1H), 1.57 (d, *J* = 6.4 Hz, 3H), 1.36 – 1.10 (m, 3H).

^{13}C NMR (mixture of *E* and *Z* isomers): (101 MHz, Acetone-d_6) δ 168.3, 168.0, 158.2, 158.0, 157.0, 156.9, 154.6, 153.5, 137.0, 136.7, 132.7, 132.5, 132.2, 132.1, 131.0, 130.4, 121.9, 115.0, 114.2, 114.1, 103.5, 103.3, 72.0, 71.9, 70.4, 70.1, 60.5, 56.1, 56.0, 55.7, 37.5, 37.4, 35.8, 28.5, 20.8, 18.7, 18.4, 15.3, 15.1, 14.5.

HRMS calc *m/z* $[C_{20}H_{22}O_6NCl]^+$: 408.1214, found 408.1210.

Oxime Stereochemistry

Oximes were isolated and assayed as mixtures of *E*/*Z* isomers. While these isomers could be enriched (and in some cases, fully resolved) chromatographically, long-term NMR and LC/MS

stability studies of enriched batches of compound 5 in DMSO-$_{d6}$ indicated slow isomerization in favor of the *E*-isomer over time. No evidence of hydrolyzed radicicol was observed in these stability experiments.

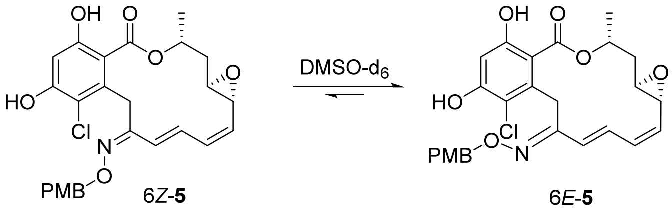

Starting Sample	Timepoint	% *E* isomer	% *Z* isomer
**5, *E*-enriched**	**Day 0**	**58%**	**42%**
	**Day 16**	**85%**	**15%**
**5, *Z*-enriched**	**Day 0**	**35%**	**65%**
	**Day 16**	**67%**	**33%**

While we chromatographically obtained isomer-enriched samples of some oxime mixtures, experiments employing these enriched samples are confounded by the use of DMSO as vehicle (due to the slow equilibration). All attempts to ascertain differences in Hsp90 inhibitory activity among the enriched samples have thus far been ambiguous, with roughly equipotent activities observed across the enriched samples. Because of these observations, combined with our inability resolve the exact oxime stereochemistry crystallographically at the obtainable resolution (one isomer was arbitrarily modeled into the structure, while the other also fits the experimental data well) we do not wish to over-speculate as to any isomeric preference for inhibition among the oxime mixtures.

Yeast Strain Construction

ScLC3827: To express wild type *C. albicans HSP90* in *S. cerevisiae,* plasmid pLC868 (pAG424-*C. albicans HSP90*) was transformed into ScLC3048 and transformants were selected on YNB supplemented with leucine, tryptophan, and adenine. The presence of pLC868 was verified by PCR using oLC3723 and 3441. Subsequently, ~10e7 cells were plated on 0.1% 5-FOA YPD agar plates to counter-select for the pKAT6 vector. An absence of pKAT6 was verified by PCR with oLC11 and oLC1869. Colonies were also verified for the correct auxotrophy by patching on YNB supplemented with leucine, tryptophan, adenine, and uracil as well as by a lack of growth on plates lacking uracil.

ScLC5036: To express *C. albicans HSP90*^{T12Q} in *S. cerevisiae,* plasmid pLC1003 (pAG424-*C. albicans HSP90*^{T12Q}) was transformed into ScLC3048 and transformants were selected on YNB supplemented with leucine, tryptophan, and adenine. The presence of pLC1003 was verified by PCR using oLC3723 and 3441. Subsequently, ~10e7 cells were plated on 0.1% 5-FOA YPD agar plates to counter-select for the pKAT6 vector. An absence of pKAT6 was verified by PCR with oLC11 and oLC1869. Colonies were also verified for the correct auxotrophy by patching on YNB supplemented with leucine, tryptophan, adenine, and uracil as well as by a lack of growth on plates lacking uracil.

ScLC5037: To express *C. albicans HSP90*^{L130AF131Y} in *S. cerevisiae,* plasmid pLC1004 (pAG424-*C. albicans HSP90*^{L130AF131Y}) was transformed into ScLC3048 and transformants were selected on YNB supplemented with leucine, tryptophan, and adenine. The presence of pLC1004 was verified by PCR using oLC3723 and 3441. Subsequently, ~10e7 cells were plated on 0.1% 5-FOA YPD agar plates to counter-select for the pKAT6 vector. An absence of pKAT6 was verified by PCR with oLC11 and oLC1869. Colonies were also verified for the correct auxotrophy by patching on YNB supplemented with leucine, tryptophan, adenine, and uracil as well as by a lack of growth on plates lacking uracil.

ScLC5038: To express *C. albicans HSP90* ^{K158ST162R} in *S. cerevisiae,* plasmid pLC1005 (pAG424-*C. albicans HSP90*^{K158ST162R}) was transformed into ScLC3048 and transformants were selected on YNB supplemented with leucine, tryptophan, and adenine. The presence of pLC1005 was verified by PCR using oLC3723 and 3441. Subsequently, ~10e7 cells were plated on 0.1% 5-FOA YPD agar plates to counter-select for the pKAT6 vector. An absence of pKAT6 was verified by PCR with oLC11 and oLC1869. Colonies were also verified for the correct auxotrophy by patching on YNB supplemented with leucine, tryptophan, adenine, and uracil as well as by a lack of growth on plates lacking uracil.

Plasmid Construction

pLC1003: This plasmid is based on pLC868 but harbors T12Q mutation in *C. albicans* Hsp90. This mutation was introduced by site-directed mutagenesis with primers oLC5120 and oLC5121. Successful introduction of the desired mutation was sequence verified with oLC611, oLC755, oLC321, oLC322, and oLC23.

pLC1004: This plasmid is based on pLC868 but harbors L130A and F131Y mutations in Hsp90. These mutations were introduced by site-directed mutagenesis with primers oLC5122 and oLC5123. The successful introduction of the desired mutation was sequence verified with oLC611, oLC755, oLC321, oLC322, and oLC323.

pLC1005: This plasmid is based on pLC868 but harbors K158S and T162R mutations in Hsp90. These mutations were introduced by site-directed mutagenesis with primers oLC5124 and oLC5125. The successful introduction of the desired mutation was sequence verified with oLC611, oLC755, oLC321, oLC322, and oLC323.

Cloning and purification of recombinant *C. albicans* Hsp90 for biochemical studies

Total RNA was isolated from log phase yeast culture using Trizol reagent and RT-PCR was used to obtain cDNA. *C. albicans* Hsp90 was amplified from *C. albicans* cDNA using the following primers: 5'GCATCCTCGAGATGGCTGACGCAAAAGTTG 3' (forward primer) and

5'GCACGCCATGGTTAATCAACTTCCATAGCAG 3' (reverse primer). Amplified product was cloned in pRSET-A vector as a 6x-His tag fusion protein and transformed into *E. coli* DH5α competent cells. Positive clones were confirmed by restriction digestion. For CaHsp90 protein purification, His-tagged CaHsp90 was expressed in *E. coli* RIL strains. Cells were grown at 37°C until the optical density of the culture reached 0.6. Induction was done with 0.5 mM IPTG at 30°C for 3 hours. Cell pellet was lysed in buffer containing 50 mM Tris-Cl pH 8.0, 1 mM imidazole, 2 mM PMSF. Protein was purified to homogeneity using Ni-NTA column.

K_d determination for ATP binding using fluorescence spectroscopy

The binding affinity of ATP to *C. albicans* Hsp90 was determined based on the property of intrinsic tryptophan fluorescence quenching by ligands. Briefly, purified Hsp90 (20 µg/ml) was incubated with varying concentrations of ligand, ATP (100 µM-3000 µM) in binding buffer (40 mM HEPES-KOH buffer pH 7.4, 5 mM $MgCl_2$ and 100 mM KCl). Intrinsic tryptophan fluorescence was measured by scanning the emission spectrum from 300-400 nm at excitation at 280 nm. For Kd calculations, the intensity at λ_{max} 340 nm was selected. Difference in intrinsic fluorescence of protein alone and in presence of ATP was plotted against ATP concentration. GraphPad Prism 5.0 software was used to analyze the binding curve and non-linear regression analysis was done assuming single-site specific binding.

Pharmacological studies

The sensitive and specific detection of CMLD013075 in biological media was accomplished by LC/MS analysis of acetonitrile extracts using a QExactive Orbitrap instrument coupled to a Dionex Ultimate UPLC system (Thermo Scientific). All determinations, both standards and experimental samples were performed in duplicate by the Whitehead Institute Metabolomics core facility. For plasma stability testing, compound (1 µM) was incubated at 37 °C in K-EDTA anticoagulated plasma from CD-1 mice (Innovative Research) for periods of time ranging from 0 to 180 mins.

Duplicate aliquots (10 μL) at each time point were extracted with 40 μL of ice cold acetonitrile and shaken for 30 min at 4 °C. The acetonitrile solvent was spiked with 10 nM imatinib as an internal standard for mass spectrometry. Following extraction, samples were spun at 20,000 x g for 15 min and de-proteinated supernatants stored at -80 °C until analysis. Standard curves were prepared in plasma matrix and processed in parallel with the experimental samples. Microsome stability testing was performed in a similar manner except that incubation of compounds was performed in CD1 mouse liver microsomes (10 mg/ml, XenoTech, Cat #M1500) supplemented with an NADPH-regenerating system (XenoTech, Cat#K5000). Plasma pharmacokinetic studies in mice were performed under a protocol approved by the MIT Committee on Animal Care (CAC). CMLD013075 was formulated in cremophor EL vehicle (Sigma, #C5135) and administered via subcutaneous injection. At intervals post injection, cohorts of 3 mice were euthanized by CO_2 inhalation and whole blood collected by cardiac puncture into K-EDTA tubes. Samples were kept on ice until plasma was separated by centrifugation and stored at -80 °C until extraction and analysis as described above for plasma stability testing.
