# Supplementary material for: Structural basis for species-selective targeting of Hsp90 in a pathogenic fungus
Source: Nat Commun. 2019 Jan 24;10:402. doi: 10.1038/s41467-018-08248-w (PMC6345968; doi:10.1038/s41467-018-08248-w)
Supplement: Supplementary file 3 — Reporting Summary [file 41467_2018_8248_MOESM3_ESM.pdf]

nature research

Corresponding author(s): Cowen, Leah E.

Reporting Summary

Nature Research wishes to improve the reproducibility of the work that we publish. This form provides structure for consistency and transparency in reporting. For further information on Nature Research policies, see Authors & Referees and the Editorial Policy Checklist.

Statistical parameters

When statistical analyses are reported, confirm that the following items are present in the relevant location (e.g. figure legend, table legend, main text, or Methods section).

n/a	Confirmed	
☐	☒	The exact sample size (*n*) for each experimental group/condition, given as a discrete number and unit of measurement
☐	☒	An indication of whether measurements were taken from distinct samples or whether the same sample was measured repeatedly
☐	☒	The statistical test(s) used AND whether they are one- or two-sided<br>*Only common tests should be described solely by name; describe more complex techniques in the Methods section.*
☒	☐	A description of all covariates tested
☒	☐	A description of any assumptions or corrections, such as tests of normality and adjustment for multiple comparisons
☐	☒	A full description of the statistics including central tendency (e.g. means) or other basic estimates (e.g. regression coefficient) AND variation (e.g. standard deviation) or associated estimates of uncertainty (e.g. confidence intervals)
☒	☐	For null hypothesis testing, the test statistic (e.g. *F*, *t*, *r*) with confidence intervals, effect sizes, degrees of freedom and *P* value noted<br>*Give P values as exact values whenever suitable.*
☒	☐	For Bayesian analysis, information on the choice of priors and Markov chain Monte Carlo settings
☒	☐	For hierarchical and complex designs, identification of the appropriate level for tests and full reporting of outcomes
☒	☐	Estimates of effect sizes (e.g. Cohen's *d*, Pearson's *r*), indicating how they were calculated
☐	☒	Clearly defined error bars<br>*State explicitly what error bars represent (e.g. SD, SE, CI)*

Our web collection on statistics for biologists may be useful.

Software and code

Policy information about availability of computer code

Data collection

For structural studies, diffraction data were collected using home X-ray sources for the apo and ADP structures. A synchrotron X-ray source (beamline 08ID-1 at the Canadian Light Source (CLS) was used for radicicol and CMLD013075 complexes. Beamline 23ID-D at APS - Argonne National Laboratory was used for SNX-2112 and AUY922 complexes.

Data analysis

For dose-response and binding affinity measurements, data were analyzed using GraphPad Prism 7 software. For crystallographic studies, data were processed with HKL3000(2,3). Structures were determined by molecular replacement using the yeast Hsp90 NBD (PDB code 1AH8)(4) as a search model for the first complex structure of the C. albicans Hsp90 NBD. This structure was used as the model for subsequent structures. Phaser(5) was used for molecular replacement calculations, while model building was performed with COOT6 and structures were refined with Phenix(7). The radicicol and SNX-2112 complex crystals were twinned, and xtriage (as implemented in Phenix)(8) identified the following merohedral twin law h,-k,-l and a twin fraction of ~0.3 for both crystals. The positions of adl compounds in the reported C. albicans Hsp90-ligand complex crystal structures were unambiguous as indicated by their simulated annealing OMIT maps (Figure S10). CMLD013075 coordinates and geometry restraints were created within the ELBOW. The stereochemistry of both models was checked by MOLPROBITY(9). Relevant data collection and refinements statistics are shown in Table S1. Protein structure superpositions were calculated with LSQKAB(10) as implemented in CCP4(11). Protein structure figures were generated using UCSF Chimera package(12). C. albicans and human Hsp90 AUY922 2D interaction diagrams (Figure S4b bottom panels) were generated using Maestro (Schrödinger Release 2016-1: Maestro, Schrödinger, LLC, New York, NY, 2016).
References:
2. Otwinowski, Z. & Minor, W. Processing of X-ray diffraction data collected in oscillation mode. Methods Enzym. 276, 307–326 (1997).
3. Kabsch, W. Processing of X-ray snapshots from crystals in random orientations. Acta Crystallographica Section D: Biological

Crystallography 70, 2204–2216 (2014).
4. Prodromou, C., Roe, S. M., Piper, P. W. & Pearl, L. H. A molecular clamp in the crystal structure of the N-terminal domain of the yeast Hsp90 chaperone. Nat Struct Biol 4, 477–482 (1997).
5. McCoy, A. J. et al. Phaser crystallographic software. J. Appl. Crystallogr. 40, 658–674 (2007).
6. Emsley, P. & Cowtan, K. Coot: model-building tools for molecular graphics. Acta Crystallogr. D. Biol. Crystallogr. 60, 2126–2132 (2004).
7. Adams, P. D. et al. PHENIX: a comprehensive Python-based system for macromolecular structure solution. Acta Crystallogr. D. Biol. Crystallogr. 66, 213–221 (2010).
8. Zwart, P. H., Grosse-Kunsteleve, R. W. & Adams, P. D. Characterization of X-ray data sets. CCP Newsl. 42, (2005).
9. Davis, I. W. et al. MolProbity: all-atom contacts and structure validation for proteins and nucleic acids. Nucleic Acids Res. 35, W375-83 (2007).
10. Kabsch, W. A solution for the best rotation to relate two sets of vectors. Acta Crystallogr. Sect. A 32, 922–923 (1976).
11. Winn, M. D. et al. Overview of the CCP4 suite and current developments. Acta Crystallogr. D. Biol. Crystallogr. 67, 235–242 (2011).
12. Pettersen, E. F. et al. UCSF Chimera--a visualization system for exploratory research and analysis. J. Comput. Chem. 25, 1605–1612 (2004).

For manuscripts utilizing custom algorithms or software that are central to the research but not yet described in published literature, software must be made available to editors/reviewers upon request. We strongly encourage code deposition in a community repository (e.g. GitHub). See the Nature Research guidelines for submitting code & software for further information.

Data

Policy information about availability of data

All manuscripts must include a data availability statement. This statement should provide the following information, where applicable:

- Accession codes, unique identifiers, or web links for publicly available datasets
- A list of figures that have associated raw data
- A description of any restrictions on data availability

The coordinates for structures and their structure factors have been deposited with the Protein Data Bank (http://www.pdb.org). PDB accession codes are provided in Table S1.

Field-specific reporting

Please select the best fit for your research. If you are not sure, read the appropriate sections before making your selection.

☒ Life sciences ☐ Behavioural & social sciences ☐ Ecological, evolutionary & environmental sciences

For a reference copy of the document with all sections, see nature.com/authors/policies/ReportingSummary-flat.pdf

Life sciences study design

All studies must disclose on these points even when the disclosure is negative.

Sample size	For all microbiological and cell culture experiments, the number of biological replicates performed was chosen on the basis of experimental feasibility and standard microbiological testing procedures established in the lab for the specific assays performed
Data exclusions	No data were excluded
Replication	All attempts at replication were successful.
Randomization	No specific method was used to randomize the allocation of mice to experimental groups for pharmacokinetic drug level measurements. Age matched female mice were injected with compound and euthanized at the time points indicated in Figure S10c prior to blood collection and processing.
Blinding	No blinding was performed

Reporting for specific materials, systems and methods

Materials & experimental systems

n/a	Involved in the study
☐	☒ Unique biological materials
☐	☒ Antibodies
☐	☒ Eukaryotic cell lines
☒	☐ Palaeontology
☐	☒ Animals and other organisms
☒	☐ Human research participants

Methods

n/a	Involved in the study
☒	☐ ChIP-seq
☒	☐ Flow cytometry
☒	☐ MRI-based neuroimaging

Unique biological materials

Policy information about availability of materials

Obtaining unique materials
Heat-shock reporter cell lines (C. albicans and human) are available from the corresponding author upon request with payment of shipping costs by requester.

Antibodies

Antibodies used
Antibodies used in this study were part of commercially available ELISA kits (Figure 7c):
R&D Systems: TNF-alpha DuoSet (DY410) and IL1-1 beta/IL-1F2 DuoSet (DY401).

Validation
The commercial kits provide optimized capture and detection antibody pairings with recommended concentrations for use. Kits have been used extensively with 168 citations listed for the IL1-beta kit and 299 citations for the TNF-alpha kit.

Eukaryotic cell lines

Policy information about cell lines

Cell line source(s)
ATCC

Authentication
The cell lines were not authenticated for the purposes of this study because their specific identity is non-critical to the conclusions drawn

Mycoplasma contamination
The cell lines were tested for mycoplasma contamination and found negative

Commonly misidentified lines
(See ICLAC register)
Cell ines are not listed in the database

Animals and other organisms

Policy information about studies involving animals; ARRIVE guidelines recommended for reporting animal research

Laboratory animals
Female 4-6 week old 129 sv/jae were used for pharmacokinetic study

Wild animals
Provide details on animals observed in or captured in the field; report species, sex and age where possible. Describe how animals were caught and transported and what happened to captive animals after the study (if killed, explain why and describe method; if released, say where and when) OR state that the study did not involve wild animals.

Field-collected samples
For laboratory work with field-collected samples, describe all relevant parameters such as housing, maintenance, temperature, photoperiod and end-of-experiment protocol OR state that the study did not involve samples collected from the field.
